# Therapeutic Drugs and Devices for Tackling Ocular Hypertension and Glaucoma, and Need for Neuroprotection and Cytoprotective Therapies

**DOI:** 10.3389/fphar.2021.729249

**Published:** 2021-09-17

**Authors:** Najam A. Sharif

**Affiliations:** Global Alliances and External Research, Ophthalmology Innovation Center, Santen Inc., Emeryville, CA, United States

**Keywords:** glaucoma, intraocular pressur, aqueous humor, neurodegenaration, neuroprotection, pharmacology, drug discovery

## Abstract

Damage to the optic nerve and the death of associated retinal ganglion cells (RGCs) by elevated intraocular pressure (IOP), also known as glaucoma, is responsible for visual impairment and blindness in millions of people worldwide. The ocular hypertension (OHT) and the deleterious mechanical forces it exerts at the back of the eye, at the level of the optic nerve head/optic disc and lamina cribosa, is the only modifiable risk factor associated with glaucoma that can be treated. The elevated IOP occurs due to the inability of accumulated aqueous humor (AQH) to egress from the anterior chamber of the eye due to occlusion of the major outflow pathway, the trabecular meshwork (TM) and Schlemm’s canal (SC). Several different classes of pharmaceutical agents, surgical techniques and implantable devices have been developed to lower and control IOP. First-line drugs to promote AQH outflow via the uveoscleral outflow pathway include FP-receptor prostaglandin (PG) agonists (e.g., latanoprost, travoprost and tafluprost) and a novel non-PG EP2-receptor agonist (omidenepag isopropyl, Eybelis^®^). TM/SC outflow enhancing drugs are also effective ocular hypotensive agents (e.g., rho kinase inhibitors like ripasudil and netarsudil; and latanoprostene bunod, a conjugate of a nitric oxide donor and latanoprost). One of the most effective anterior chamber AQH microshunt devices is the Preserflo^®^ microshunt which can lower IOP down to 10–13 mmHg. Other IOP-lowering drugs and devices on the horizon will be also discussed. Additionally, since elevated IOP is only one of many risk factors for development of glaucomatous optic neuropathy, a treatise of the role of inflammatory neurodegeneration of the optic nerve and retinal ganglion cells and appropriate neuroprotective strategies to mitigate this disease will also be reviewed and discussed.

## Introduction

Human and animals heavily rely on good vision to perform their daily tasks and for survival, and indeed eyesight is undoubtedly the most valuable of our precious senses. The eyes being windows for the brain is apt since 80% of the external information reaching the neural networks comes in from the visual system. Thus, visual impairment in any form has a devastatingly negative impact on most people whose greatest fear is blindness. Sadly, the World Health Organization (WHO, 2019) reports that due to increasing poverty, poor nutrition, pollution, smoking, reduction in natural resources, diminishing supply of affordable basic hygiene and healthcare products at a global level and lack of timely diagnosis, the incidence of poor vision and blindness continues to rise. Unsurprisingly, Africa, Asia and South America represent the nations where this situation continues to worsen. However, ready supply of rich foods and a rising tide of obesity in the developed countries also is burdening healthcare systems and increasingly causing a rise in ocular disorders and diseases. Development of cataracts and other refractive errors such as myopia account for the major causes of blindness on our planet. However, the incidence of glaucoma, an optic neuropathy comprising several different forms, is the second leading cause of blindness worldwide affecting nearly 80 million patients and which is expected to debilitate >112 million by 2040 (Tham et al., 2014; Flaxman et al., 2017). More than 195 million suffer from age-related macular degeneration (AMD; both wet and dry forms) and 145 million from diabetic retinopathy. Contextually, the estimated societal economic burden imposed by visual impairment/blindness just in the United States is >$16 billion/annum when accounting for decreased quality of life, disability, morbidity and lost overall productivity. With an increasingly aging world population, ophthalmic disorders represent a rising healthcare issue of huge proportions worldwide (WHO, 2019).

## Architecture of the Anterior Chamber of the Eye

**FIGURE 1:**
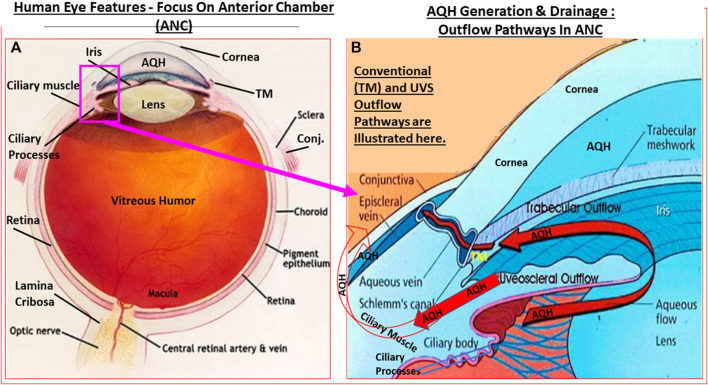
Outline of the basic overall anatomy of the human eye illustrating some of the key features discussed in the text. **(A)**. In (B), the key elements of the AQH synthetic machinery (ciliary procesess), and AQH outflow via the trabecular meshwork (TM conventional outflow) and via the uveoscleral pathway from the anterior chamber are shown. Note: none of the elements shown are to scale.

**FIGURE 2:**
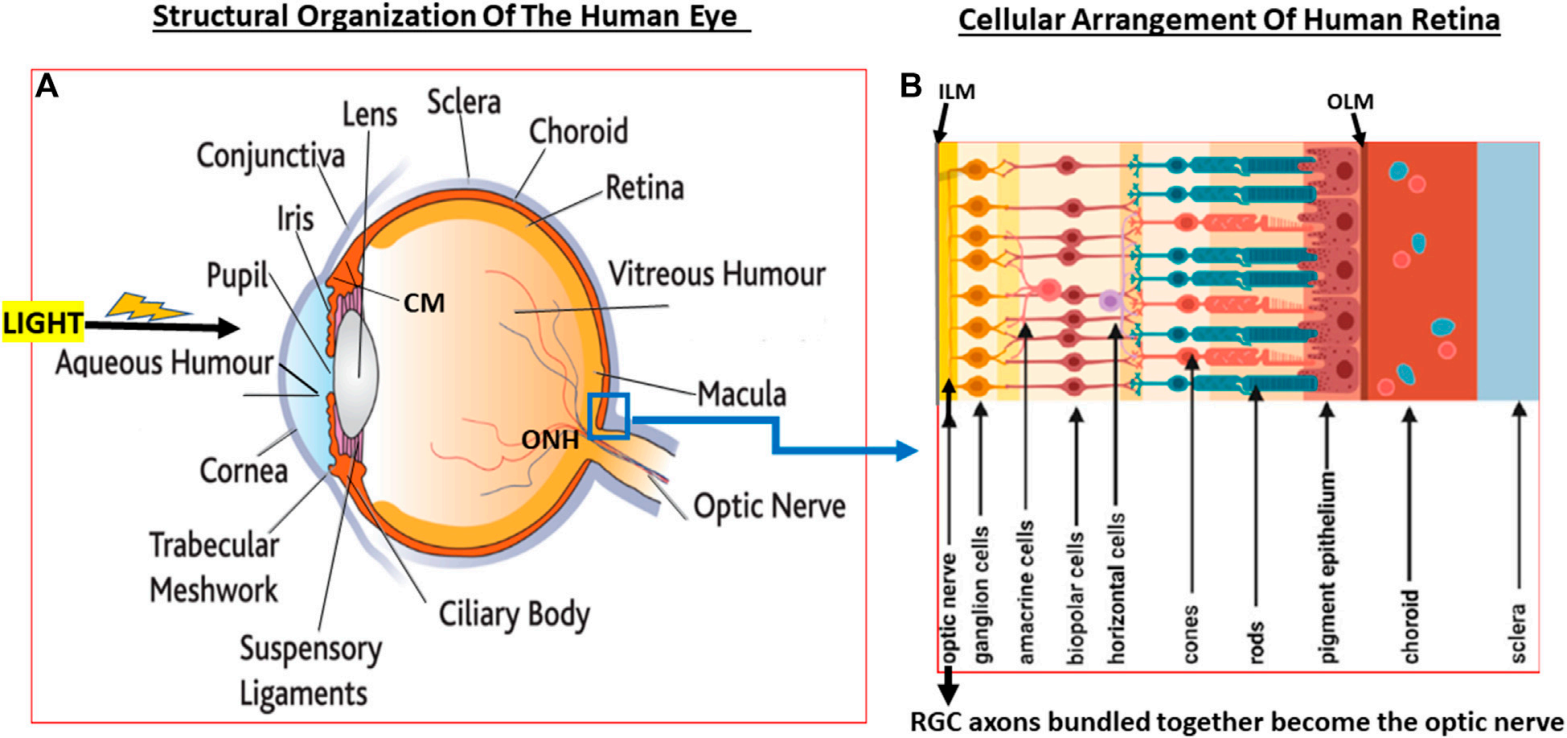
A pictorial view of the eye structures **(A)** with special reference to retinal architecture showing the various types of cells and their relative positions **(B)**.

Before delving into the pathological basis of glaucoma, it is necessary to describe the anatomical structures and the functions of the visual axis. The mammalian eye is exposed to the outside world somewhat unprotected although the eye socket offers some protection. Similarly, the protective thick white fibrous outer layer (sclera) of the eyeball affords the eye its shape while insulating the interior components from possible damage. The transparent cornea at the front of the eye consists of five layers of different cell types and represents a specialized scleral tissue (DelMonte and Kim, 2011). The conjunctiva is an extension of the lateral parts of the cornea and sclera but it is highly vascularized (unlike the cornea) and ends up as the tissue lining the underside of the eyelids (Weingeist, 1973). Sitting inside a capsular “pocket” and suspended by ligaments is the lens inside the eye a few centimeters behind the corneal endothelial cell layer. Suspended in front of the lens is the iris that forms the pupil where the iris sphincter muscle contracts or relaxes to regulate the amount of light passing to the lens. Just lateral to the lens ligaments is the ciliary body (CB; Smith, 1973) composed of the ciliary processes (CP)/ciliary epithelium (CE) and the ciliary muscle (CM) which is attached to the lens to allow accommodation (Figures 1A,B, 2A,B). The space between the lens and the cornea is the anterior chamber of the eye which is filled with a watery solution (aqueous humor [AQH]) which is produced by the CP/CE of the CB (Civan and Macknight, 2004). Together with the sclera and cornea (and the vitreous humor in the posterior chamber behind the lens [see ahead]), the AQH helps maintain the overall shape of the eyeball.

The AQH also nourishes the cells lining the ANC of the eye as it percolates through the ANC to exit via the angular space between cornea and iris via a filtration system, trabecular meshwork (TM), that is connected to the Schlemm’s canal (SC) that allows the AQH to pass into the veinous circulation via series of complex plexi of smaller vessels (Figures 1A,B, 2A,B). Under normal physiological conditions the amount of AQH produced (∼2 µL/hour) by the non-pigmented cells of the CE equals the amount draining from the ANC, and thus homeostasis is achieved. Recent single cell-based transcriptome research has revealed that at least eight different types of cells reside within the TM/SC outflow pathway and as many as seven sub-types within the UVS pathway in human and monkey eyes with mice and pigs showing similar expression patterns (van Zyle et al., 2020). The filtration area was comprised of two-types of TM cells (expressing known marker genes such as *MYOC, MGP* and *PDPN*), SC cells and resident macrophages. However, the structural elements of the TM were composed of Schwalbe’s line cells (TM-beam cells; two-types expressing gene markers *FABP4* and *TMEFFs*) in the non-filtering area of the TM around the juxtacanicular region. The juxtacanicular cells differentially expressed various genes including *CH13L1*, *ANGPTL7, RSPO4, FMOD* and *NELL2*. Interestingly, SC cells displayed a profile made up of both lymphatic endothelia and blood macrophages. Lastly, there were differences in the genetic expression profiles of TM cell-types (e.g., *MYOC, FOXC1, PITX2, CYP1B1, LOXL1, ANGPT1* and *EFEMP1*) and SC cells (e.g., *CAV1, CAV2, TEK, PRSS23, ANGPT2*), with differences extending to genes involving OHT-related high IOP *vs* controls. Differences were also observed in RGCs genes which were unrelated to elevated IOP. Therefore, the collective studies from several groups highlight the fact IOP generation and regulation are mediated by a complex array of cell-types in the outflow pathways, and that these elements appear to be well conserved across multiple species Liton et al., 2009; Liu et al., 2016; Kizhatil et al., 2014; Stamer et al., 2015; Sathiyanathan et al., 2017; Carnes et al., 2018; Patel et al., 2020; van Zyl et al., 2020).

Additional single-cell transcriptomic investigations of human ANC cells revealed up to twelve distinct cell-types (Patel et al., 2020). Of the TM cells, myoblastic and fibroblastic signatures were obtained yielding a Schwann cell and macrophagic profile of genes. In contrast, the SC cells exhibited a more lymphatic/blood vasculature genetic signature (Patel et al., 2020). These features correlate well with the ability of TM cells to contract/relax (Wiederholt et al., 2000), and their ability to phagocytose cellular debris, ECM and chemical agents (Alvarado et al., 1981; Alvarado et al., 1984; Grierson and Howes, 1987; Matsumoto and Johnson, 1997; Sherwood and Richardson, 1988), and for the SC pathway cells to drain away the filtered AQH to the veinous circulation (Thomson et al., 2014; Bernier-Latmani and Petrova, 2017; Acott et al., 2020).

In contrast, the posterior segment of the eye behind the lens is filled with a jelly-like material (vitreous humor [VH]), and this chamber is substantially larger than the ANC. The VH, composed of many different proteins and water, also helps in shaping the eyeball and also provides much cushioning and protection to the retina that lines the inside of sclera at the back of the eyeball, and it probably is also involved in removing some of the waste products of the retina. Just as the cornea at the front of the eye is composed of many cell layers, the retina is highly complex and contains many specialized cell-types.

## Architecture of the Retina and Optic Nerve

Since glaucoma results from structural and functional failures at multiple levels throughout the visual system, it is important to be aware of the composition and organization of the various components. In the rear of the human eyeball, the retina contains many different types of cells that are also essentially transparent such that light reaches the back of the eye and penetrates to the deeper layers of the retina where the photo-sensitive cells (photoreceptors; rods and cones) reside. Whilst cones are concentrated in a central region of the retina called fovea, the rods are mostly located in the peripheral regions of the retina. Whereas the cones are specialized for high acuity tasks like reading and color perception, rods are responsible for night vision and respond best to dim light (Grossniklaus et al., 2015).

**FIGURE 3:**
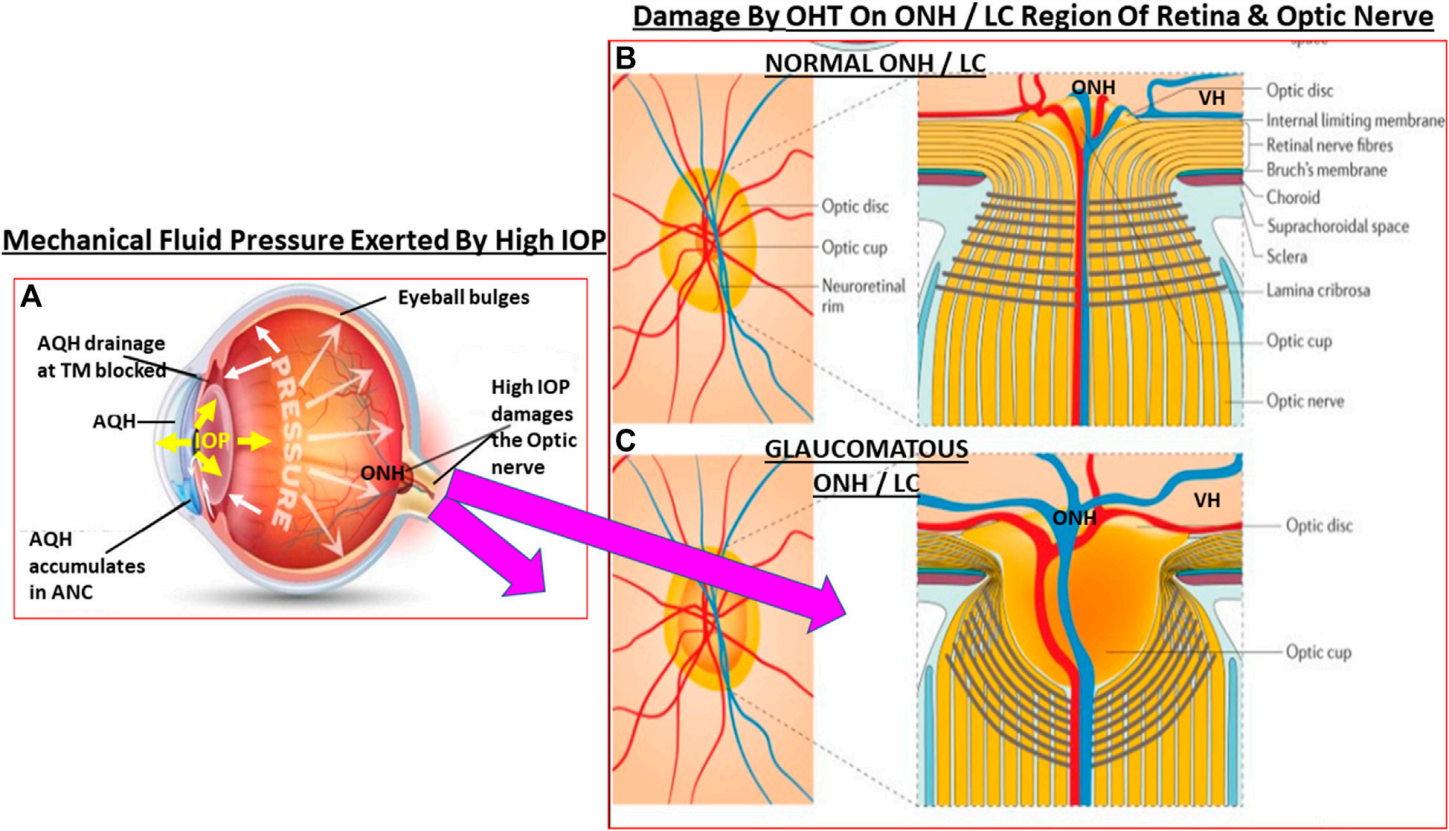
This figure depicts the effects of ANC fluid pressure (IOP) being radiated out to all parts of the eyeball. In OHT the elevated IOP **(A)** grossly and deleteriously affects the weak areas of the posterior globe at the level of the ONH/LC. The normal structural integrity of the LC and ONH area yields a small optic cup to optic disc ratio **(B)**. When the LC/ONH tissues are damaged due to mechanical stress-induced remodeling, the LC area becomes excavated and the optic cup enlarges leading to a significantly increased cup to disc ratio **(C)**. Additionally, the RGC axons are reduced and the retinal vasculature becomes displaced and causes potential ischemic conditions in the retina.

The jelly-like VH fills the majority of the posterior segment of the eye. At the back of the eye, the VH is separated from the retinal tissue by the inner limiting membrane. Next comes a multilayered retinal nerve fiber layer (RNFL) composed of the axons of the RGCs that converge at the optic nerve head (ONH)/optic disc area (Herrera et al., 2019), pass through a delicate tissue (lamina cribosa; LC; Daguman and Delfin, 2018; Figures 3A,B) at and behind ONH and are bundled together to form the optic nerve that exits the eyeball. Behind the RGCs (Russo et al., 2016; Detwiler 2018) are several layers of interneurons comprising bipolar, amacrine and horizontal cells followed by the photo-sensitive photoreceptors (rods and cones) and then the retinal pigment epithelial (RPE) cells (Figures 1A,B, 2A,B) (Vecino et al., 2016; Grünert and Martin, 2020). Muller glial cells run the full length of the retina anteriorally from the RPE cells to the RGCs. The RPE cells are seperated from the capillaries of the choroidal circulation by the outer limiting membrane (Bruch’s membrane) (Figures 1A,B, 2A,B). Each optic nerve travels to the optic chiasm and crosses over to reach the contralateral area of the thalamic brain nuclei. The latter process the information and send it on to the visual cortex thereby conveying the visual signal information from the RGCs to the brain.

**FIGURE 4:**
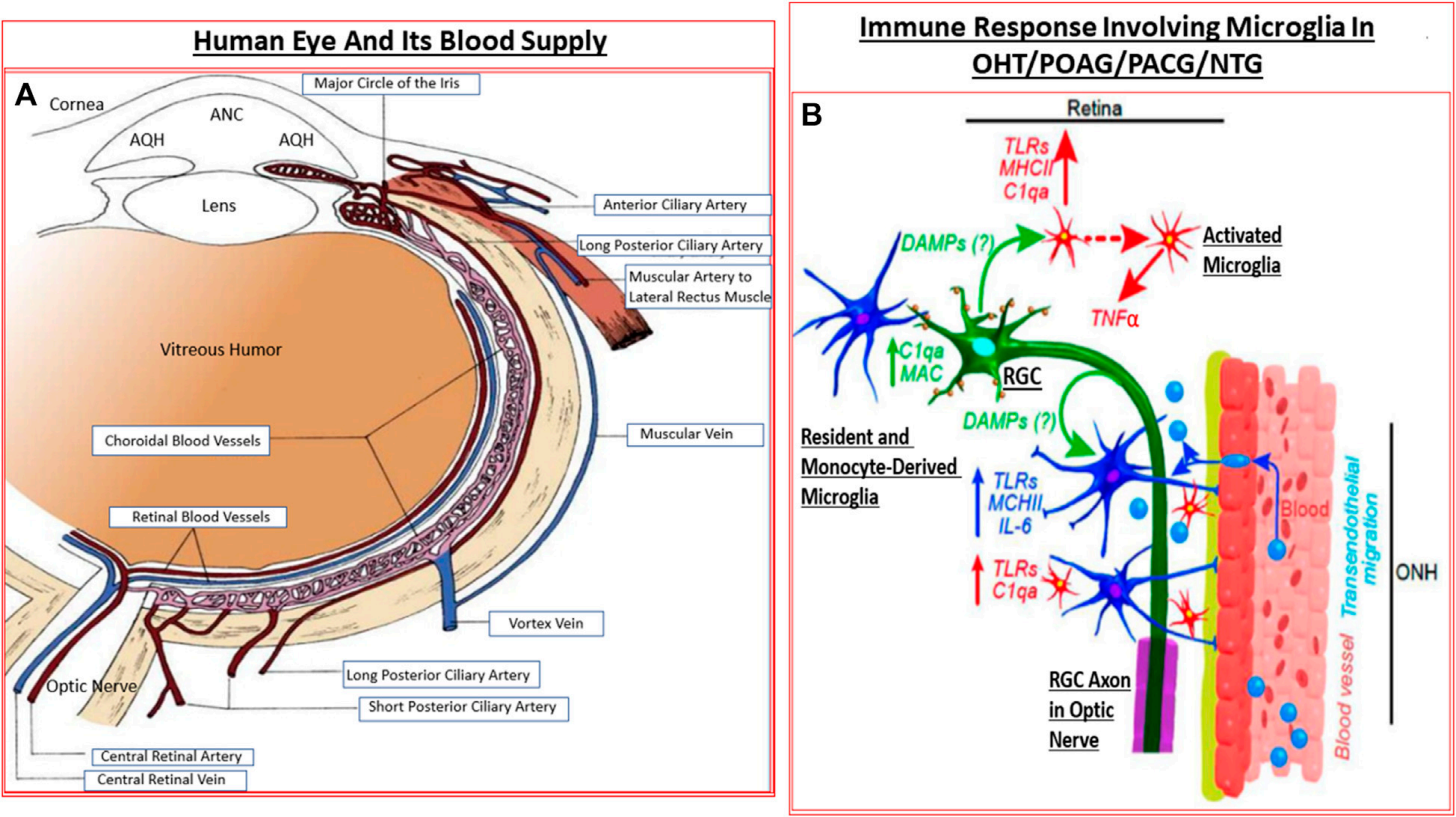
The detailed anatomical location and distribution of ocular blood vessels **(A)**, and the interplay between the resident cells in the retina and blood-borne immune cells infiltrating into the retina **(B)** under GON conditions are shown. The activated microglia and astrocytes elaborate various injurious cytokines and up-regulate TLRs resulting in inflammatory neurodegeneration.

The retina is a high energy-demand tissue and it receives nutrients and oxygen from the retinal arteries and the choroid (Kiel 2010; Ansari and Nadeem, 2016). The blood supply to the eye originates from the internal carotid artery as the ophthalmic artery whose branches include the central retinal artery, the short and long posterior ciliary arteries, and the anterior ciliary arteries. The central retinal artery (CRA) bends many times before reaching the optic disc and it, together with the short posterior ciliary arteries provide blood supply to the retina. The CRA travels in or beside the optic nerve as it enters the sclera at the back of the eye, and from where it then branches out to supply the layers of the inner retina which are closest to the inner limiting membrane/VH. The central retinal vein and vortex veins collect the venous blood (Kiel 2010; Ansari and Nadeem, 2016). The latter veins merge with the inferior and superior ophthalmic veins that drain into the pterygoid venous plexus and the facial vein. The retinal venules and veins merge into the central retinal vein (CRV) which exits the eye with the optic nerve parallel and counter-current to the CRA (Kiel, 2010; Ansari and Nadeem, 2016) (Figure 4A).

The dura, arachnoid and the pial membrane sheaths encase the optic nerve (Anderson, 1973) which is composed of three zones referenced relative to the LC. While collaterals from the choroid and retinal circulation supply the prelaminar zone (i.e., inside the eye relative to the LC), short posterior ciliary and pial arteries supply the laminar zone. Lastly, the post laminar zone is supplied by the pial arteries. Venous drainage occurs via the CRV and the pial vein (Kiel, 2010; Ansari and Nadeem, 2016) (Figure 4A). For the optic nerve vessels, the laminar zone marks the transition from exposure to the IOP to the cerebral fluid pressure (intracranial fluid pressure; ICFP) within the optic nerve sheath.

## The Physiological Aspects of Vision

Much joy and happiness comes to us through our eyes as we appreciate the beauty around us, especially the display mother nature arranges for us. It is no wonder then that eyesight is so precious.

At a macrolevel, vision occurs when light entering the cornea passes through the pupil and is focused by the lens onto the retinal photoreceptors. Here, many complex biochemical and electrical signals are created and processed as neurotransmitters are released at synapses and they activate receptors/ion-channels to relay the information within 0.1–0.2 milliseconds. Thus, as the photoreceptors absorb the light, Na^+^-channels are closed to allow intracellular levels of Na^+^ ions to build-up and the cell membrane potential becomes positive. Now, the Ca^2+^-channels are closed, and inhibitory transmitter levels are decreased which removes the inhibition and the bipolar cells are stimulated/excited creating an actional potential in the RGCs which are sent down their axons to the thalamic brain nuclei. Defects in the membrane repolarization process and locally produced endothelin impairs axonal transport and deprives RGCs of neurotrophin support from the brain (Stokely et al., 2002; Fischer et al., 2019).

The RGCs are separated from the VH by a thin transparent inner limiting membrane. Neurons in the inner two retinal layers exhibit complex receptive fields that allow them to detect small changes in contrast reflecting shadows or edges of objects. The RGCs collect all this array of information, including color, and transmit it to the brain down their axons, which are bundled together to form the optic nerve. The optic nerves from each eye cross over at the optic chiasm as they traverse to the first brain relay station, the lateral geniculate nucleus (LGN) of the thalamus (Liang et al., 2018; Masri et al., 2020). The optic nerve also provides input to the pretectum (that controls pupillary response to light) and to the superior colliculus (SC) which is responsible for moving the eyes in response to ambulatory signals. The information about the image is passed from the thalamus to the visual cortex for final decoding and visual perception (Kravitz et al., 2013). Signal transmission from the retina to the brain occurs in approximately 0.15 s, and information transfer happens at roughly 10 million bits per second and vision occurs.

As described above, most optic nerve projections from the retina traverse to the LGN which sorts retinal signals into parallel streams containing color, structure, motion and contrast. Since the LGN is also structurally layered, its top four parvocellular layers handle color and fine structure, while the bottom two magnocellular layers process contrast and motion. Not surprisingly, the primary visual cortex is also highly organized to receive LGN inputs to maintain fidelity and complexity of the information being sent to it. The LGN magnocellular and parvocellular layer cells send long axons to the ancient part of the brain comprised of the primary visual cortex (PVC) (Masri et al., 2020). Here in the V1 region of the PVC, the cells are arranged in complex ways which permit the visual system to assign the objects being perceived in a spatially precise manner. Specifically, these V1-PVC cells are organized so that a direct mapping of RGCs is essentially and precisely imprinted in the visual cortex on a point-by-point basis in a columnar pattern of connections alternating between the left and right eyes. This allows the V1 area of PVC to position objects perceptually in the horizontal and vertical axes. Rapid comparison of the signal inputs from the two eyes by V1-PVC cells allows perception of depth of vision thereby rendering the images into three-dimensions. As V1 cells sharpen the lines and edges of images, the cells of V2 region of PVC refine the coloration of the object images. Color and form perception in V3 and V4 regions of PVC, inferior temporal lobe recognition of face and object, and motion and spatial awareness in the parietal lobe of the cortex cover the majority of the permutations of visual perception. The above illustrates how intricate and yet efficient and strong the visual system is in so many animals and humans. Such is the wonderous tale of light entering the eye and the miracle of seeing the outside world around us. It is therefore imperative that we do not take sight for granted and do everything possible to preserve and cherish it. By actively researching into the causes of vision loss and finding suitable treatment modalities, we can all try to give the gift of sight to those unfortunate people in our communities who are afflicted with visual impairment.

## Various Forms of Glaucoma

Focusing on the ANC of the eye will now permit a detailed description and discussion about ocular hypertension (OHT) and glaucoma. This ocular disease is represented by a group of pathological conditions which all culminate in the death of RGCs and eventual loss of much of their axonal connections to the brain structures mentioned above (Weinreb and Khaw, 2004; Quigley, 2011; Weinreb et al., 2014; Jonas et al., 2017; Sharif, 2017; Bhandari et al., 2019). The disease is most often associated with elevated intraocular pressure (IOP), and results in gradual loss of peripheral vision in the early stages, with eventual impact on central vision before irreversible blindness if left undiagnosed and untreated. Sadly >50% of the patients who eventually learn about their glaucoma were unaware of their ocular condition WHO, 2019). Diagnostic imaging includes stereoscopic photos and optical coherence tomography (OCT) of the optic nerve for characteristic structural changes at the retinal nerve fiber layer (RNFL), while visual field testing helps to detect functional changes in vision (Quigley, 2011; Weinreb et al., 2014; Jonas et al., 2017; Sharif, 2017; WHO, 2019; Burton et al., 2021).

Many different forms of glaucoma are known (Zukerman et al., 2020). The most common form is primary open-angle glaucoma (POAG) where the angle between the cornea and the iris is normal but the drainage of the AQH via the TM/SC outflow pathway is slowed down or is blocked, and where the uveoscleral (UVS) outflow pathway is poorly operational and results in elevated intraocular pressure (IOP). In primary closed-angle glaucoma (PACG; Sun et al., 2017; Chan et al., 2019; Wang et al., 2019), the iris is displaced and it obstructs the AQH egress from the ANC to rapidly raise IOP (Gazzard et al., 2003) (Figures 1A,B). Both these forms of glaucoma cause OHT by elevating the IOP which then damages the retina and the optic nerve at the back of the eye. In normotensive glaucoma (NTG; Mallick et al., 2016), which is very common in Japan, the eye pressure is in the normal range (14–21 mmHg) but the patient continues to lose vision due to other deleterious factors (Collaborative Normal-Tension Glaucoma Study Group, 1998a; Collaborative Normal-Tension Glaucoma Study Group, 1998b). In some newborn children the ANC eye pressure is abnormally elevated and this leads to congenital glaucoma.

Secondary glaucomas, as the name implies, occurs due to an ocular insult or due to another medical problem such as ANC inflammation (causing uveitic glaucoma) or due to steroid-induced accumulation of complexed extracellular matrix proteins in the TM that prevent AQH efflux, for example. Neovascular glaucoma occurs when abnormal growth of new blood vessels obstructs the AQH drainage pathways in the ANC and it is usually caused by diabetes or high blood pressure. Pigment dispersion syndrome (pigmentary glaucoma) results from aberrantly released melanin granules from the iris which then occlude the AQH drainage pathways thereby causing OHT. Myopic young white men are prone to pigment dispersion syndrome than other people. The most well known animal model of pigmentary glaucoma is the DBA/2J mouse and its variants (Williams et al., 2013; Cooper et al., 2016; Harder et al., 2020). Lastly, there is exfoliation glaucoma that occurs in some patients who have exfoliation syndrome (Ritch et al., 2003; Anderson et al., 2018), a disorder that causes detachment of cells and other debris from the ANC to clog the TM/SC which blocks AQH fluid from draining, thereby raising the IOP. Recent research shows that exfoliation glaucoma may have a genetic linkage to disease (Aung et al., 2018).

Inflammatory and immunogenic elements produce cardinal signs and symptoms of uveitis, including in uveitic glaucoma (Kwon et al., 2017). Anterior uveitis includes inflammation of the iris and ciliary body within the ANC of the eye and can account for up to 90% of all uveitic episodes reported by patients and healthcare professionals. This disorder can be episodic or chronic in nature. Intermediate uveitis, also known as pars planitis, consists of vitritis—vitreous cavity cell inflammation due to deposition of inflammatory cells in the vitreous. Regardless, combined uveitic disease is the 3^rd^ largest cause of blindness worldwide, and glaucoma associated with uveitis is a very serious disease requiring immediate attention since the IOP can rise suddenly and to a high magnitude (Kwon et al., 2017).

The elevated IOP caused by uveitis and the IOP-spikes associated within these acute or chronic episodes of intraocular inflammation can cause rapid damage to the optic nerve unless the high IOP is reduced quickly and then maintained at a relatively low level (Siddique et al., 2013). As mentioned above, since systemic inflammation and abnormal immune responses can greatly contribute to uveitic glaucoma, the patient requires attention by both an ophthalmologist and a rheumotologist. Glaucoma surgery is usually required to rapidly reduce the elevated IOP, coupled with immunosuppression and/or treatment with corticosteroids to prevent visual impairment and eventual vision loss. Non-infectious posterior uveitis, which is rarer than the anterior uveal uveitis, can now be treated using intravitreal injection of sirolimus, an immunosuppresant that inhibits the mammalian target of rapmycin (mTOR) (Nguyen et al., 2011). Such treatment modalities may be useful for treating uveitic glaucoma once the initially high IOP has been reduced somewhat. Likewise, in a form of pigmentary glaucoma model (DBA/2J mouse model), it appears that lengthening of the nodes of Ranvier in the optic nerve and redistribution of Na^+^-channel precede axonal transport deficits and eye-brain signaling (Pease et al., 2000; Ito and Di Polo, 2017; Crish and Calkins, 2011; Crish et al., 2010; Crish et al., 2013), features that parallel changes seen early on in multiple sclerosis (MS) axonopathy (Smith et al., 2018; McGrady et al., 2020). These deficits could be abrogated by a week of systemic immunosuppressant therapy with fingolimod, a sphingosine-1-phosphate receptor agonist, a drug that is used in relapsing-remitting multiple sclerosis disease, thus offering another therapeutic approach to combat optic nerve damage due to pigment accumulation in the ANC of the eye that prevents AQH drainage and results in OHT. Mycophenolate, sustained release corticosteroids (Borkar et al., 2017), other pharmacological agents, trabeculectomy and AQH drainage shunts (Kwon et al., 2017), along with new generation biologics (Thomas et al., 2019) provide much hope for patients who succumb to non-infectious posterior uveitis, and uveitis-associated OHT/glaucoma (Kesav et al., 2020).

If we focus on the two chief forms of glaucoma impacted by direct features and events in the ANC of the eye, we can summarize the major risk factors associated with POAG and PACG. Thus, decades of research have concluded that POAG risks factors include: elevated IOP (ocular hypertension, OHT), low intracranial fluid pressure, low retinal perfusion, advanced age, African-Caribbean-Latin American ancestry, family history of glaucoma, thin corneas, myopia, diabetes, high blood pressure and low diastolic pressure. PACG occurs suddenly when the iris is displaced and blocks the AQH drainage pathway causing a rapid elevation of IOP, perhaps as high as 70 mmHg. Its clinical manifestations include nausea, blurred vision, ocular pain, cloudy corneas and halos around lights. The risk factors linked to PACG include: advanced age, shallow ANC angles, Asian-Eskimo ancestry, family history of PACG, hyperopia and female gender.

Several clinical trials conducted in the late 1900s early 2000s revealed that lowering of the IOP is highly beneficial and directly slows down and can prevent glaucoma progression in most forms of glaucoma (GLT, 1995; Collaborative Normal-Tension Glaucoma Study Group, 1998a; Collaborative Normal-Tension Glaucoma Study Group, 1998b; AGIS, 2001; Lichter et al., 2001; Heijl et al., 2002; Kass et al., 2002; Gordon et al., 2002; Leske et al., 2007). Consequently, clinical medicine has focused on lowering and controlling IOP to help preserve sight of patients with glaucoma and these aspects will be discussed ahead. However, glaucoma etiology is complex and a number of pathophysiological events converge to induce RGC death and RGC axonal loss: elevated IOP (OHT), retinal ischemia, oxidative stress in the TM/SC and retina, neurotrophin and energy deprivation, and toxicity due to locally elevated levels of glutamate, endothelin, cytokines, nitric oxide (and perhaps carbon monoxide), and proteases (Bucolo and Drago, 2011; Fu and Sretavan, 2012; Evangelho et al., 2019) (Figure 4B). These aspects will be discussed below.

## Accumulated Aqueous Humor Dynamics in the anterior chamber (ANC) Related to Primary Open-Angle Glaucoma

**FIGURE 5:**
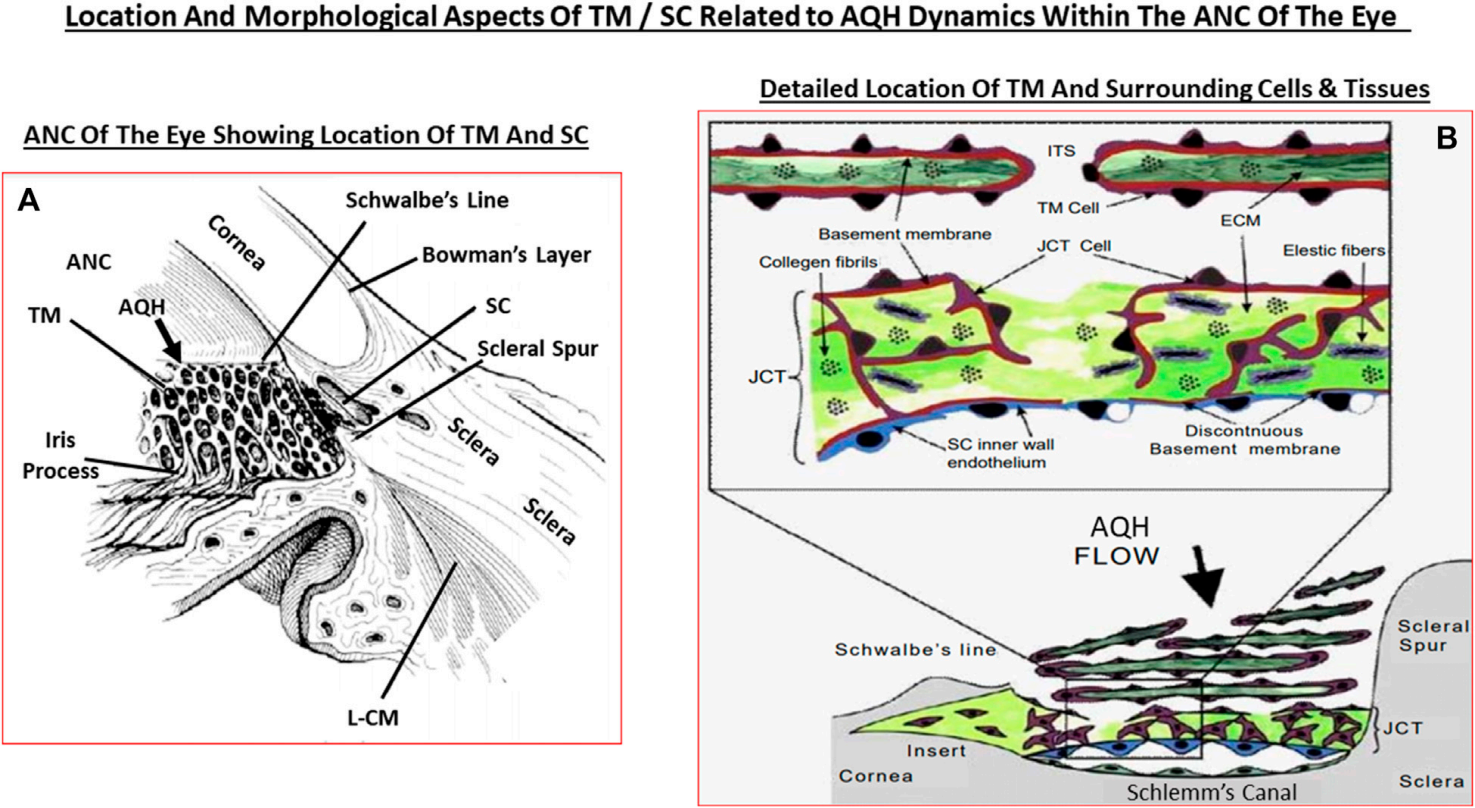
This figure shows the ANC and the location of the TM and SC relative to the cornea, iris and the ciliary body **(A)**, and the more detailed juxtaposed cellular features of the TM and the SC in relation to the AQH flow **(B)**.

Since OHT due to excess AQH accumulation in the ANC is the root cause of POAG, this requires a detailed appraisal. As mentioned earlier, the CE (mainly non-pigmented ciliary epithelial cells [NPCECs]) of the CP within the CB generate the clear AQH fluid by passive diffusion, ultrafiltration and secretion, with the latter being the predominant event (reviewed by Civan and Macknight, 2004). Since the ANC is avascular, the AQH flowing through the ANC has very important functions that provide a nurturing (provision of O_2_, glucose and growth factors) and a stable environment (achieved by balancing nutrient provision and removal of waste products) for the cells/tissue lining the ANC (Kaufman, 2020). As shown in Figure 1B, POAG begins most frequently with an imbalance of AQH production by the CE and its drainage from the ANC of the eye via the conventional (TM/SC) and unconventional (uveoscleral, UVS) outflow pathways (Acott et al., 2020; Wang et al., 2020). TM outflow of AQH accounts for 70–90% and UVS outflow for 10–30% of total AQH drainage from the ANC. Research has shown that TM/SC-mediated AQH efflux is pressure-dependent, and that IOP is mainly due to the back-pressure generated due to blockage of this system (Acott et al., 2020; Wang et al., 2020). Since AQH ultimately exits the eye via collector channels of the TM/SC system and empties into the intrascleral venous plexus, deep-scleral plexus, and into episcleral vessels and thus the veinous circulation, the total resistance within this collective group of channels/plexi/blood vessels causes the elevation of IOP (Thomson et al., 2014; Kaufman, 2020). A closer look at the TM/SC structures (Abu-Hassan et al., 2014; Figures 5A,B) illustrates that several different cell-types coexist and that the majority of the resistance to AQH outflow from the conventional system occurs at the innermost uveal region, the middle corneal scleral region and the outermost juxtacanalicular tissue which essentially becomes obstructed during development of OHT/POAG.

**FIGURE 6:**
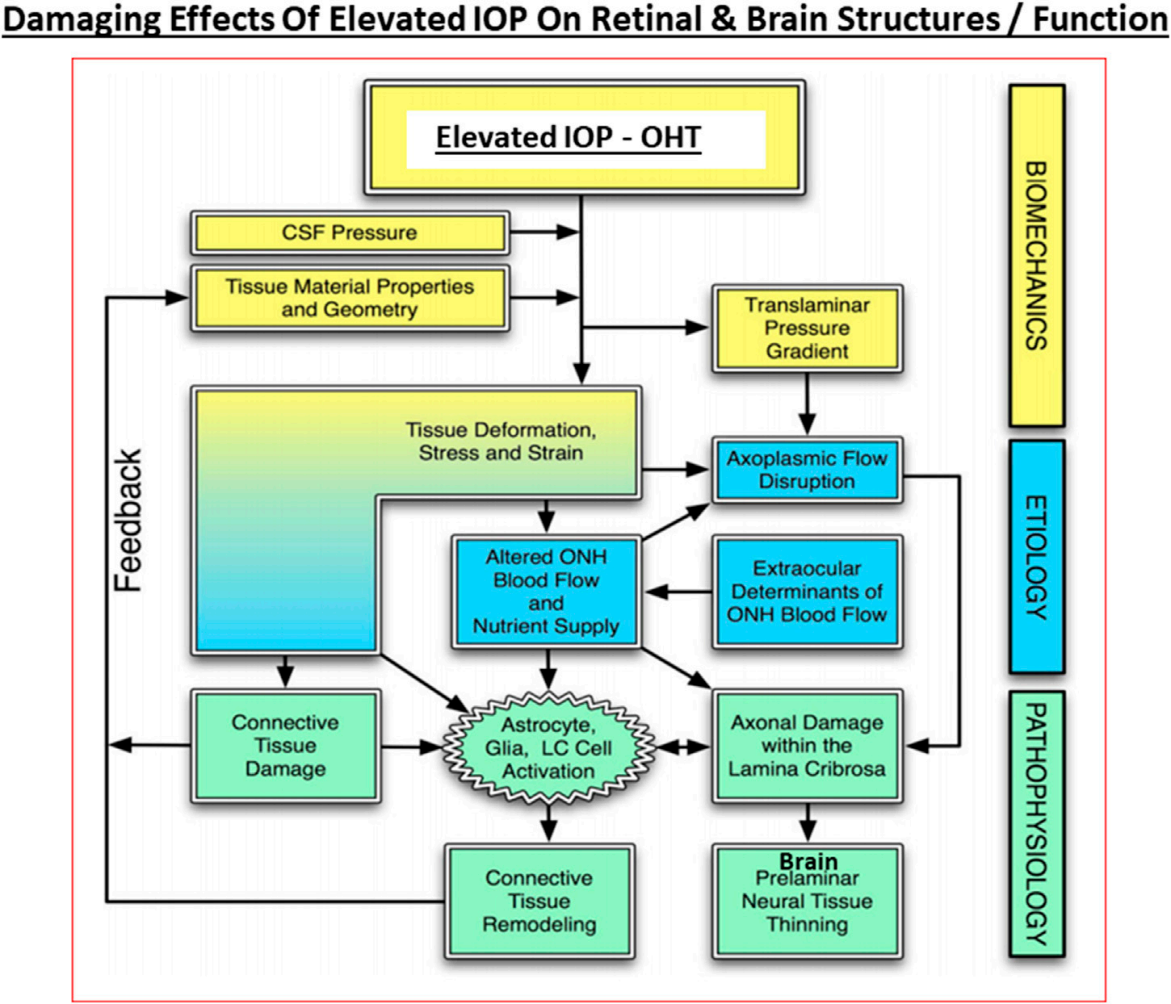
The interplay between the biomechanical fluid pressure-induced stress from the ANC due to elevated IOP, the etiological elements (e.g., reduced axonal transport and ischemia), and the final pathological features and end-points observed in POAG and other forms of glaucoma at the retinal/LC/ONH and brain levels are depicted here.

Under normal circumstances, especially in young eyes, the TM cells are abundant and actively phagocytose cellular debris and other accumulating substances such as collagen and fibronectin within the extracellular matrix (ECM; De Groef et al., 2016) to keep the AQH flow constant as the TM filters the AQH in a relatively passive manner. The SC endothelial cells appear to actively transport AQH by generating pores across their surfaces, and perhaps via aquaporins, thereby helping AQH exit the ANC (Acott et al., 2020; Wang et al., 2020). During aging and due to other local deleterious events and processes and ECM accumulation, the number of TM cells decreases as does their remaining phagocytic/autophagic activity (Alvarado et al., 1981; Alvarado et al., 1984; Grierson and Howes, 1987; Matsumoto and Johnson, 1997; Sherwood and Richardson, 1988). Thus, in normal eyes, AQH generation is maintained at a constant rate and a stable IOP is achieved by changes in regulation of AQH outflow/alteration of resistance in the outflow pathway. Normal average IOP is 15 mmHg and >90% of human subject have IOPs between 10 and 21 mmHg. However, in POAG, it is the AQH outflow pathway that is compromised (due to ECM/cellular debris accumulation due to increased transforming growth factor-β (TGF-β) (Tripathi et al., 1994; Hasenbach et al., 2016; Rao and Stubbs, 2021), aberrant cross-linking of ECM which stiffens the TM cells (Yemanyi et al., 2020), possible defects in the SC pore/aquaporin system (Wang et al., 2020), increased resistance at the SC due to angiopoietin/Tie-2 pathway defects (Thomson et al., 2014; Thomson et al., 2019; Bernier-Latmani and Petrova, 2017) and the IOP continues to rise due to the constant addition of AQH to the ANC from the CB. It is estimated that the veinous blood vessel component of the AQH drainage system accounts for <25–50% of total outflow resistance whereas the juxtacanalicular region of the TM/SC area is the major contributor providing >50–75% of total outflow resistance in POAG. However, the resistive impact of distal AQH drainage vessels on IOP probably increases due to their constriction under pathological conditions due to bloodborne and/or AQH-borne vasoconstrictor mediators such as reactive O_2_ species, thromboxane and endothelin. Even though TM/SC cells, which have endothelial cell morphology and physiology, appear to show an upregulated nitric oxide (NO) synthase and release the vasodilator gaseous transmitter NO under pressure to compensate for the increased resistance (Schneemann et al., 2003), these measures may be insufficient to decrease the IOP and these elements may overall be substantially down-regulated in glaucomatous ANC. Furthermore, there may be a deficiency of MMPs being released locally around the TM/SC resulting in aberrantly diminished tissue remodeling and ECM accumulation (De Groef et al., 2016). Also, as the IOP changes during the day and night, IOP spikes and general fluctuations are more common in glaucomatous eyes than in normotensive eyes (Bengtsson et al., 2007; Caprioli and Coleman, 2008; Siddique et al., 2013; Kim and Caprioli, 2018). It is believed that such irregular eye pressure changes are highly detrimental to the visual system, and since they occur mostly at night, it is imperative that these IOP fluctuations (Jasien et al., 2019) are diminished as much as possible in order to de-stress the vulnerable elements of the retina/optic nerve at the back of the eye in the quest to protect the RGCs and their axons from this glaucomatous optic neuropathy (GON) (Sharif, 2017, 2018a,b) (Figure 6).

## Retinal/Optic Nerve Components Participating in Glaucoma Pathogenesis

As noted above, even though the AQH dynamics imbalance in the anterior segment of the eye is responsible for onset of POAG/PACG, the whole visual system saccumbs to the overall disease. Again, in order to better understand how glaucomatous damage occurs, it is important to be aware of the anatomy of the retina and the optic nerve.

The retina is a highly specialized structure that possesses a diverse population of cells ranging from photoreceptors, interneurons, retinal ganglion cells (RGC; both pigmented and non-pigmented; Cruz-Martín et al., 2014; Sanes and Masland, 2015; Baden et al., 2016; Russo et al., 2016; Detwiler, 2018) and contain three sub-types of glial cells which serve a diverse range of functions. Glial cells were thought to be mainly structural components of the retina (Figures 2A,B). However, cell profiling has revealed distinct physiological and morphological differences amongst them. Indeed, microglia, Muller cells (Bringmann et al., 2006) and astrocytes are not passive bystanders in the etiology of GON. Astrocytes are star-shaped and appear similar to microglia though they are significantly larger than the latter cells. Astrocytes are fairly stable in their quiscient state and are homeostatic in their role, maintaining blood-brain/blood-retina barriers, regulating blood flow, recycling neurotransmitters, and maintaining synaptic connections (Liu and Neufeld, 2003; Hernandez et al., 2008; Williams et al., 2017c). Astrocytes perform these roles through contacts with capillaries, neuronal cell bodies and RGC axonal bundles in the rear of the retina and at the optic nerve level. Sensing trauma or injury as from elevated IOP-induced deformation of the ONH, astrocytes release glial fibrillary acid protein (GFAP) (Inman and Horner, 2007). This results in the formation of a scar that attempts to isolate the site of injury. Additionally, the astrocytes chronically release various cytokines which upregulate different genes and proteins needed in summoning help and repairing the surrounding tissue (Liu and Neufeld, 2003; Hernandez et al., 2008; Williams et al., 2017c) (Figure 6).

Muller cells appear to be the true benefactor cells of the retina and they are exclusive to the retina, being quite populous (Bringmann et al., 2006). Their cell bodies are located in the inner nuclear layer, but they extend their processes throughout the retinal layers to ensure contact with the majority of the resident cell-types they serve (Figure 2B). Under normal circumstances, Muller glia retain their morphology and physiology helping to maintain a stable local cellular environment in the retina in terms of provision of nutrients, maintaining ionic strength and pH (Reichenbach and Bringmann, 2013; Goldman, 2014; Lust and Wittbrodt, 2018). These cells also provide neurotrophins to neurons and phagocytose local debris. They also ingest and recycle unused local neurotransmitters and toxic waste, and they support and maintain rods, cones and RPE cells. As with astrocytes, Muller glia change their morphology and functions in the wake of deleterious conditions within the retina such as hypoxia or inflammation. They then begin releasing GFAP and upregulate Toll-like-receptors (TLRs), and start secreting cytokines and chemokines thereby signaling trauma to the eye surveillance system(s) (Reichenbach and Bringmann, 2013; Goldman, 2014; Lust and Wittbrodt, 2018).

Microglia represent the CNS-related macrophages that are quiet surveyors of the local extracellular environment (Chen et al., 2002) (Figure 4B). Beginning as monocytes, they enter the CNS during early development and differentiate into resident microglia that spread out throughout the brain, spinal cord and the retina/optic nerve. They check on the health of the neighboring cells as they move around. In the visual system, these quiscent microglia become activated when they sense ischemia, pathogenic infection, aberrant cell death in the retina, at the ONH and/or the optic nerve (Neufeld, 1999), and along with disturbances of the synaptic connections within the brain relay nuclei (Lam et al., 2009; Ebneter et al., 2010). The activated microglia (Yuan and Neufeld, 2000; Yuan and Neufeld, 2001) now release nitric oxide (NO) (Neufeld, et al., 1997), cytokines and chemokines which cause local vasodilation and allow monocytes to infiltrate and transform into more reactive glia thereby amplifying the inflammatory/immunological response to injury/trauma in the visual axis (Neufeld, 1999; Chen et al., 2002; Bosco et al., 2008; Bosco et al., 2011; Wei et al., 2019; Rashid et al., 2019) (Figure 4B). TLRs, major histocompatibility complex (MHC) recognition molecules and complement activation also ensues (Borucki et al., 2020).

Oligodendrocytes and Schwann cells represent additional neuroglia relevant to the retinal-brain axis. The function of the Schwann cells is to produce layers of insulating myelin that encases each RGC axon, and provision of nutritients and structural support to the Schwann cells and the axons befalls on the oligodendrocytes (Fitzner et al., 2011). In this manner, each RGC axon becomes myelinated, except in a small area directly adjacent to the back of the eyeball where the axons exit the rear of the eyeball. Bundling of the RGC axons results in the formation of the optic nerve that crosses over at the optic chiasm and travels on to the brain thalamic relay stations. This unmylenated area of the optic nerve, together with the ONH at the LC, and also the spaces in between the thicker myelin segments along the optic nerve (nodes of Ranvier) are the most delicate components within the visual system and contribute to optic neuropathy/optic neuritis (Bastakis et al., 2019). They are susceptible to injury from intrinsic and extrinsic damaging factors and insults, including the stress and strain brought on by the mechanical forces of elevated IOP in POAG/PACG, especially IOP spikes (Siddique et al., 2013; Pan et al., 2014; Tan et al., 2018; Sherman and Cafiero-Chin, 2019). Lastly, other important retinal cells include amacrine and horizontal cells which modulate communication between photoreceptor- and the major neural cell-types, including bipolar-cells and RGCs of which many types exist (Ou et al., 2016; Vecino et al., 2016; Grünert and Martin, 2020). Early-stage OHT alters the electrical transmission of signals from the RGS to the thalamus (Bhandari et al., 2019), and this deficit increases over time (Trivedi et al., 2019).

## Ocular Hypertension/Primary Open-Angle Glaucoma and Factors Involved in Retinal/Optic Nerve Damage

Globally, the predominant form of glaucoma is POAG (currently ∼54 million suffering) which represents ∼75% of all forms of glaucoma. Primary closed-angle glaucoma (PACG) has the second highest occurrence (∼23 million patients). Unfortunately, Asian and African nations have the highest number of POAG and PCG patients, greatly outnumbering those afflicted with these glaucoma forms in Europe and North America. Close to 5 million patients are projected to have POAG in North America by 2040.

Akin to most chronic diseases, POAG has a age-related occurrence with patients being diagnosed with this disease almost exponentially between age 40 to age 80 in almost all geographical locations. POAG is asymptomatic, the patient feels no pain and is oblivious to the disease development since other warning signs are all absent. This “silent thief of sight” makes its detection and diagnosis difficult, and it is estimated that 50% of future POAG patients remain undiagnosed. Additionally, during early stages of POAG/PCG/NTG, the patient does not notice much change in the vision and the brain compensates (Bham et al., 2020) for losses of RGCs that have already occurred and are happening (Weinreb et al., 2014; Jonas et al., 2017; Sharif, 2017; Sharif, N. A. 2018). However, as the conditions progress, a tipping-point is reached where the patient notices loss of peripheral (lateral) vision and objects observed appear incomplete and/or become blurry and/or infrequent double vision occurs (Crabb, 2016). Sadly, this signals the demise of >400k RGCs and their axons in the affected eye of the patient (Calkins and Horner, 2012). Ophthalmic examination of the POAG/PCG afflicted patient, including optical coherence tomography (OCT) (Quigley et al., 1989) and visual field tests (Harwerth and Quigley, 2006; Sasaoka et al., 2008; Sponsel et al., 2014), reveal that significant damage has occurred at the ONH (Burgoyne et al., 2005) causing increased optic disc cupping (Downs et al., 2011), that the retinal nerve fiber layer (RNFL) has thinned (due to RGC axon loss) (Harwerth and Quigley, 2006; Soto et al., 2011; Yu et al., 2015; Tu et al., 2019); and scotomas (dead zones) developed in the visual field (Harwerth and Quigley, 2006; Sasaoka et al., 2008; Sponsel et al., 2014). The level of IOP increase is well correlated with optic nerve damage (Chauhan and Drance, 1992; Guo et al., 2005; Yohannan and Boland, 2017; Torres and Hatanaka, 2019) and decrease in retinal function (Parisi, 2003; Kasi et al., 2019; Torres and Hatanaka, 2019; Turkey et al., 2019). The patient’s vision loss begins to accelerate and immediate treatment is required to retard further visual impairment (Karaca et al., 2020). Initially the patient is prescribed topical ocular eyedrops containing medicine (usually a prostaglandin analog (Hellberg et al., 2001, 2002) but it may be beta-blocker like timolol, betaxolol or levobetaxolol to lower the IOP in the ANC of the eye (Weinreb et al., 2014). If the patient is unresponsive or the IOP is poorly controlled, the physician will switch patient to another type of IOP-lowering drug and may resort to adjunctive therapies to achieve the desired IOP reduction using fixed-dose combination products (Hollo et al., 2014). Lasering of the TM may be necessary to create holes for the AQH to egress from the blocked TM system (Weinreb et al., 2014; Jonas et al., 2017). Surgery may be necessary to implant an AQH shunt (Sehi et al., 2010; Pahlitzsch et al., 2017; Sadruddin et al., 2019) that drains the AQH from the ANC of the eye. Finally, if all fails the patient will undergo direct physical surgery to literally create a hole and a flap (bleb) to drain the fluid from the ANC (Bhandari et al., 1997; Vinod and Gedde, 2021). These and newer treatment modalities to treat POAG will be discussed later once the etiological aspects of POAG and GON are described in more detail.

So, even though GON eventually results from the demise of the RGCs and the elimination of the RGC-axonal connections to the brain, in most instances the disease really begins in the ANC. How the afore-mentioned cascade of events operates in OHT/POAG/NTG can be described as follows based on our current understanding. The aging processes and cellular dysfunctions brought on by mitochondrial defects (Wentz-Hunter and Ueda, 2002; He et al., 2008; Izzotti et al., 2011) causing energy deficiency within TM cells lead to their senescence. Thus, a reduced TM cell population is left to perform the AQH filtration and cleansing (Alvarado et al., 1981; Alvarado et al., 1984; Grierson and Howes, 1987; Caballero et al., 2004; Babizhayev and Yegorov, 2011). Additionally, the phagocytic (Sherwood and Richardson, 1988; Matsumoto and Johnson, 1997; Zhou et al., 1999) and autophagic activities (Munemasa and Kitaoka, 2015; Porter et al., 2015; Stothert et al., 2016; Hirt and Liton, 2017) of the remaining TM cells is also substantially reduced resulting in a build-up of ECM (Teixeira et al., 2015; Shen et al., 2020) and other cellular debris within the corneoiridial angle of the ANC of the eye (Gabelt et al., 2005). The reduced perfusion of the ANC (Johnson, 1996) causes an accumulation of cellular waste products and oxidized proteins like lipofucin, ceramide and other lipid metabolites (Aljohani et al., 2013), reactive O_2_ species (ROS; He et al., 2008), elevated levels of homocysteine and reduced anti-oxidants (You et al., 2018), elevated endothelin (Zhao et al., 2020), IL-6 and TGF-β1 in the AQH (Liton et al., 2009; Perera et al., 2016). Furthermore, miR-29b is down-regulated which increases ECM secretion (Luna et al., 2012), and additional ATP is screted (Li et al., 2012), along with stimulation of activation factor-4 all of which increase endoplasmic reticulum stress in the TM cells (He et al., 2008; Izzotti et al., 2011; Kasetti et al., 2017) causing TM cell death (Kasetti et al., 2020; Zhao et al., 2020; Ying et al., 2021). Additionally, damaged or dying TM cells release numerous deleterious compounds (e.g., nestin, A-kinase anchor protein, actin-related protein 2/3 complex, numerous miRNAs that negatively impact the ANC) which increase cell aging, TM cell apoptosis and ECM deposition (Izzotti et al., 2011) thus exacerbating the issues in the ANC. Additionally, as mentioned earlier, increased resistance at the SC (Stamer et al., 2015; Wang et al., 2020) and distal veinous drainage plexi resulting from upregulation of the angiopoietin/Tie-2 pathway (Thomson et al., 2014; Thomson et al., 2019; Bernier-Latmani and Petrova, 2017) also contributes to the overall OHT and eventual RGC death (Figure 6).

In certain cases, topical ocular dexamethasome treatment for ocular surface or ANC inflammation causes protein complexing (Yemanyi et al., 2020) and formation of cross-linked actin networks (Bermudez et al., 2017) and results in steroid-induced glaucoma. Moreover, there’s evidence that accumulation of mutant myocilin (Kasetti et al., 2016; Kasetti et al., 2021) and amyloid proteins (Orwig et al., 2012), and IL-6-induced release of TGF-β1, which promotes ECM deposition in the ANC (Liton et al., 2009) and causes secretion of endothelin (Von Zee et al., 2012), causes IOP elevation. Glaucomatous mutant myocilin suppresses autophay and activates the IL-1/NFκB inflammatory cascade within TM cells and some of them die (Itakura et al., 2015; Lynch et al., 2018). All these processes coupled with other deleterious events in the TM/SC reduces the flexibility of these structures (Borrás and Comes, 2009; Liu et al., 2016; Borrás, 2017; Wang et al., 2017) thereby reducing their strength and capacity to filter and drain the AQH from the ANC. The compromized mechanoelasticity (Overby et al., 2014; Pang, 2021) of the TM/SC structures causes adverse gene expression (Vittal et al., 2005) and further restricts AQH outflow drainage (Acott et al., 2020). The resultant elevation of IOP and IOP spikes (Asrani et al., 2000), as the eye tries to regain homeostasis, starts to expand the eyeball as the fluidic pressure distorts the ocular structural components throughout the eye, beginning at the cornea and being transmitted to the rear of the eyeball (Gottanka et al., 1997). This mechanical distortion severely stresses the fragile ONH (Hernandez et al., 1989; Burgoyne et al., 2005; Downs et al., 2011; Xu et al., 2014; Park et al., 2015; Quillen et al., 2020) and the LC (Lee et al., 2013; Daguman and Delfin, 2018) at the back of the eye where the optic nerve leaves the eyeball. Scleral stiffining at the level of the ONH/LC (Coudrillier et al., 2016) prevents the latter tissues from being able to absorb the pressure created by increased IOP and local inflammation ensues. As the eye seeks to normalize the ocular physiology, IOP spikes (Asrani et al., 2000) are generated and these are even worse than the initial OHT, causing constriction of the optic nerve axons at the LC (Hollander et al., 1995) and thus more damage to the ONH/LC tissues (Coudrillier et al., 2016). Mechanical pressure-sensitive (MP-S) cell-types such as LC cells, ONH astrocytes and peripapillary scleral cells detect the increased IOP, and perhaps also the lower intracranial fluid pressure behind the eyeball (ICFP; Berdahl et al., 2008; Wostyn et al., 2015; Jóhannesson et al., 2018; Price et al., 2020). Stretch-linked ion-channels (e.g., TRPV-1 and TRPV-4) are also aberrantly activated in these stressed cells (Ryskamp et al., 2016). Either due to presence of amyloid and/or tau-derived pore-forming peptides (Last and Miranker, 2013; Camilleri et al., 2020; Farrugia et al., 2020; Vassallo, 2021), or high levels of TGF-β2, or independent of these, mitochondral dysfunction ensues resulting in oxidative stress and excess intracellular Ca^2+^-accumulation stresses the LC cells (McElnea et al., 2011) and the local microglia are activated (Bosco et al., 2008; Bosco et al., 2011; Gauthier and Liu, 2016). The latter release MMPS, proteases, and other inflammatory cytokines and chemokines (Ha et al., 2015) which then cause remodelling of the tissue thereby weakening the LC structure. These events cause the optic nerve and the associated blood vessels to bend resulting in vasocontriction/ischemia and reducing axonal flow which eventually kills the RGCs (Martin et al., 2006). As mentioned above, similar changes occur in the ANC where TM, and perhaps the juxtacanicular cells adjoined to the SC cells, become oxidatively stressed under pressure and due to accumulated TGF-β2 (Tripathi et al., 1994; Rao and Stubbs, 2021) and release detrimental factors such as reactive oxygen molecules, endothelin, and copious amounts of NO (and perhaps carbon monoxide [CO]; Bucolo and Drago, 2011) that have toxic effects on the surrounding cells, probably damaging them or killing some of them. During such distress the compromised TM/SC cells also release more TGF-β1/2 that remodels the TM and excerbates the situation causing further increases in resistance to AQH outflow, contributing to IOP elevation in the ANC. This vicious cycle continues at the front and back of the eye and any potential regeneration of axons is suppressed by intracellular kinases and transcription factors (e.g., Chintala et al., 2015; Apara et al., 2017).

The pressure-induced TRPV-channel activation within RGCs and their axons at the ONH begins disrupting their ionic balance which rapidly drains their energy and leakage of cellular constituents such as oxidized proteins, ATP, glutamate, and other substances into the extracellular space ensues. The oxidative stress generates and causes accumulation of extracellular lipofusin, an intralysosomal, non-degradable auto-fluorescent macromolecule which reduces autophagy and clearance of the debris (McElnea et al., 2014). ONH microglia become activated and start releasing pro-inflammatory cytokines, matrix metalloproteases (MMPs)/proteases, and vasodilatory and other toxic substances such as NO (Cho et al., 2011). The upregulated cytokines (e.g., interleukin-1 [IL-1], IL-6, IL-8, tumor neurosis factor [TNFα]) and NO/CO at the ONH induce vasodilation and edema at the ONH/LC region and within the optic nerve (Figures 3A,B). Attracted by the cytokines and chemokines, monocytes and leukocytes surge into the retina and optic nerve, further amplifying the inflammatory cascade with resultant damage. Additionally, the LC tissue is weakend by the MMP-induced degradation of the supporting ECM. The weight of the RGC axons and the associated retinal blood vessels bends and further distorts the structures at the ONH/LC which leads to ischemia/hypoxia and oxidative stress (Neufeld et al., 2002; Nguyen et al., 2011), local release of endothelin causing further vasoconstriction thereby reducing retinal perfusion, and the vicious cycle continues unabated (Prassana et al., 2011). Adding insult to injury, the increased tortoucity of the RGC axons causes disruption of the axonal transport of growth factors and mitochondria from the brain back to the RGC bodies (Dengler-Crish et al., 2014; Fahy et al., 2016). The inflammatory and immunologic insults on the optic nerve components induces a dislocation of the RGC axonal terminal connections to the thalamic LGN and pretectal regions of the brain (Dai et al., 2012; Ghaffarieh and Levin, 2012; Fujishiro et al., 2020) with ensuing retrograde atrophy of the RGC axons and neuronal loss in the LGN (Yucel et al., 2000; Yucel et al., 2001; Gupta et al., 2007). Dropout of RGC axons thins the optic nerve and the RNFL at the ONH. This cascade of events negatively impacts the neurones in the brain and the retina. The decline in retinal-LGN-superior collicular connectivity results in C1q/C3, MAC and GFAP accumulation in the thalamus and visual cortex where the neurones also begin to die. RGCs whose axons had been disconnected from the LGN/pretectum spiral into apoptotic demise as their energy and trophic support diminishes and then stops. The dying RGCs empty their intracellular contents (e.g., ATP, glutamate and DAMPs) into the extracellular space. The microglia and astroctyes detect these distress signals and begin the cellular clearance mechanisms using proteases to prune RGC dendrites and activate the phagocytic/autophagic removal of dead retinal neurones. Glia fill the spaces where the RGCs and interneurones resided and patient’s vision in that region is lost. Cerebral neuronal plasticity compensates for such early losses of RGCs (Bham et al., 2020). Even though these events occur over many years, the unrelenting chronic retinal and brain inflammation/immune response cascades rob the OHT/POAG/PACG patient of additional peripheral vision as more and more RGCs die. With only 600k RGCs left of the original million, the pateint now notices significant visual disturbances amounting to diminished visual acuity, contrast sensitivity, occasional double-vision and a narrower visual field. This is a critical point in the patient’s journey living with OHT/POAG/PACG where diagnosis and treatment initiation are paramount in order to preserve eyesight in the affected eye. AQH drainage from the ANC of the eye is now urgently needed at this stage by pharmaceutical and/or surgical intervention.

Acute inflammation signals the need to initiate damage limitation and beginning of the protective remedial process once the body experiences stress, trauma or injury. However, the local and pan-immune system is activated when a chronic microbial infection and/or inflammation sets in. Numerous cell-types mediate the inflammatory and immunogenic response during the initial phase and progression of the infection(s). Historically, bacterial and viral infections led to the development of the mammalian immune system (Rowan and Taylor, 2018; Flemming, 2018; Tang et al., 2020). Subsequent adaptation of this system has permitted the sensing of cellular stress and the clearance of dead or dying cells in order recycle cellular components to conserve energy, aid neurogensis and to epigenetically increase survival. Consequently, the host defense mechanism became responsiveness to structural characteristics found in pathogens known as pathogen-associated molecular patterns (PAMPs) which microbes express (Rowan and Taylor, 2018; Flemming, 2018; Tang et al., 2020). Astrocytes and microglia possess such signal responsive mechanisms, and they also detect damage-associated molecular patterns (DAMPs, e.g., heat-shock proteins [HSPs], uric acid, αβ-crystallin and double-stranded DNA) which stressed and/or injured cells release to alert the surveillance system (Howell et al., 2007; Soto and Howell, 2014). Thus, pattern recognition receptors (PRRs; e.g., TLRs; Luo et al., 2010; Poyomtip, 2019) on microglia and astrocytes recognize PAMPs and DAMPs and initiate and mediate the inflammatory response within the stressed tissue (Zhou et al., 2005; Vernazza et al., 2020; Parsadaniantz et al., 2020) (Figure 4B) Upon activation, TLRs signal transduction initiates recruiting and stimulation of mitogen-activated protein kinase (MAPK) and kinase IκB (Luo et al., 2010; Poyomtip, 2019) which then help elaborate numerous transcription factors (e.g., NK-κ-B, AP-1, interferon-regulator factor). The latter encode and cause the production and release of inflammatory cytokines and chemokines (Yuan and Neufeld, 2000; Tezel et al., 2010; Ha et al., 2015; Wilson et al., 2015). Mannose receptor (activated by C-type lectin), purinergic receptors (e.g., P2XR7; activated by ATP released during RGC stress/lysis) (Resta et al., 2007; Hu et al., 2010; Sakamoto et al., 2015), the multiprotein unit complexes called the inflammasome, and scavenger receptor (that eliminates leaked lipids and oxidized proteins) are other important PRRs (Howell et al., 2007; Soto and Howell, 2014). The latter collectively enhance inflammation via caspase activation (and thus production of inflammatory cytokines after inflammasome activation by ATP/TLR-4/amyloid-derived peptides) (Chi et al., 2014; Yerramothu et al., 2018). These events, along with activation by mannose residues and antigen-antibody complexes, trigger the complement cascade (Duce et al., 2006; Stasi et al., 2006), the most ancient elemental aspect of the innate immunity system. C1q and C3 components of the complement system, together with the deposition of the membrane attack complex (MAC), destroy the vulnerable cells in the injured/traumatized tissue through cell lysis (Stasi et al., 2006; Howell et al., 2011; Howell et al., 2013; Howell et al., 2014; Williams et al., 2016) (Figure 4B). Activated microglial and astrocytic-derived cytokines and chemokines chemotactically recruit monocytes and leukocytes from the dilated vasculature to the site of injury, and the inflammatory response is amplified further (Yuan and Neufeld, 2000; Tezel, 2011; Tezel, 2013; Tezel et al., 2010; Ha et al., 2015; Wilson et al., 2015). Defects in the cellular debris clearance mechanism(s), autophagy, exacerbate the situation and probably cause further cellular demise (Chintala 2006; Deng et al., 2013; Lin and Kuang, 2014; Munemasa and Kitaoka, 2015; Adornetto et al., 2020). Stress and hypoxia-induced glutamate release and newly generated reactive oxygen species contribute further to the neurotoxicity within the retina and ONH area (Ju et al., 2008; Januschowski et al., 2015).

**FIGURE 7:**
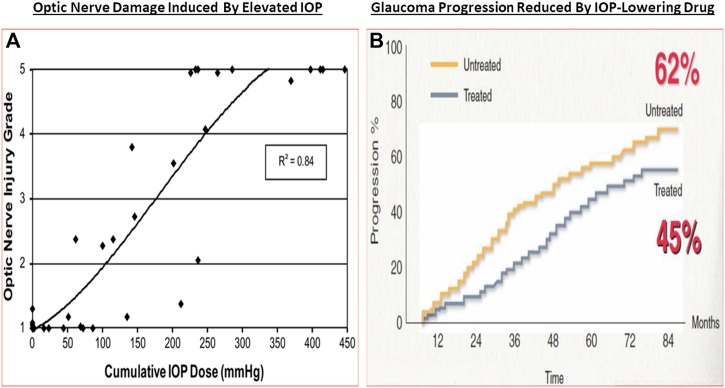
The correlation between increasing IOP and RGC loss/optic nerve damage **(A)**, and the ability of IOP-lowering drug treatment to slow down the GON/glaucoma progression **(B)** are shown in here.

The classic debate of which happens first, failed function or failed structure is a complex one. Regardless, structural changes within the optic nerve and retina precede the functional visual deficits resulting from chronic OHT, POAG/PACG and NTG. Temporally, the lag period between onset of OHT and glaucoma diagnosis contributes heavily to visual deficits, and thus preservation of vision requires the earliest possible diagnosis of these asymptomatic diseases. The strong correlation between structural damage throughout the visual system and eyesight deficits of animals and humans with glaucoma has been confirmed by numerous investigators (Quigley et al., 1989; Burgoyne et al., 2005; Weinreb and Lindsey, 2005; Harwerth and Quigley, 2006; Sasaoka et al., 2008; Downs et al., 2011; Struebing and Geisert, 2015; Torres and Hatanaka, 2019). Similarly, many studies have demonstrated a progessive optic nerve damage due to increasing IOP in OHT animal eyes (e.g., Figure 7A), and indeed several clinical trials have demonstrated that IOP reduction delays glaucoma progression (reviewed in Weinreb et al., 2014) (Figure 7B). The multiplicity of factors and events mediating the pathogenesis of OHT/POAG/PACG/NTG appear to be reproduced to a large extent in animal models of these ocular disorders as described above.

**FIGURE 8:**
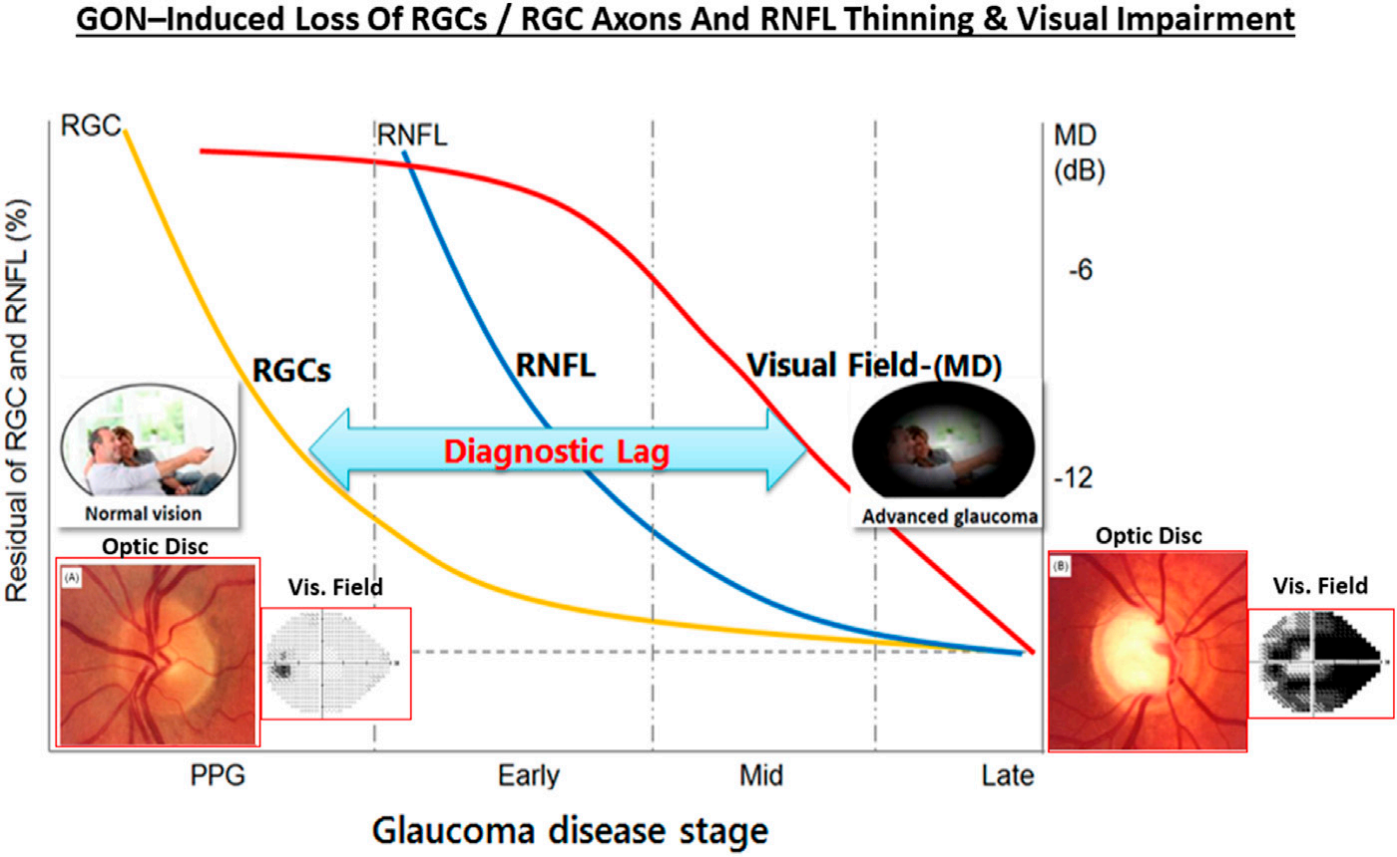
The relationship between OHT, GON, RGC and RGC axonal loss, RNFL thinning and visual impairment over time is pictorially shown here. The insets depict the optic disc and the visual field as the GON progresses.

One intriguing possibility that has some experimental support is that GON has an autoimmunogenic component in addition to the IOP-induced component (Wax and Tezel, 2009; Tezel, 2011; Tezel, 2013; Wax 2011; Geyer and Levo, 2020). Thus, significantly altered antibody directed against ocular cells/tissues were detected in serum, AQH and retinal samples of glaucoma patients, observations that were reproduced in IOP-independent animal models of glaucoma. As IgG autoantibodies switch-on the complement system, C1q/C3 and MAC complexes were found in retinas of OHT rodents, OHT primates in glaucoma patients. Similarly, anti-heat-shock-protein (HSPs) antibodies to HSP-27 and HSP-60 were found in sera of glaucoma and NTG patients (Wax, 2011; Grus et al., 2006; Grus et al., 2008). Additionally, high circulating levels of antibodies to glutathione-S-transferase, gamma-enolase and alpha-fodrin were also detected in these patients (Kremmer et al., 2001; Joachim et al., 2005; Joachim et al., 2007; Joachim et al., 2008; Joachim et al., 2012; Joachim et al., 2014; Grus et al., 2006; Grus et al., 2008). Even though the latter autoantibodies are pathogenic, others like beta-crystallin and vimentin are protective, and these appear to be down-regulated in GON. Antibodies to myelin basic protein and IgG-antibodies were found in the retinas of POAG, NTG and pseudoexfoliation glaucoma patients suggesting that a generalized autoimmune response against visual cell/tissue components can occur (Joachim et al., 2007a,b; Grus et al., 2008; Hammam et al., 2008). Clearly, much more research is needed to find and confirm such etiological aspects of the immune response in OHT and various forms of glaucoma (Gramlich et al., 2013; Joachim et al., 2014; Gramlich et al., 2016; Joachim et al., 2013; Skonieczna and Grabska-Liberek, 2014; Beutgen et al., 2019). Nevertheless, it is clear that age-related and/or pathological events occurring at the ANC-level (i.e., OHT) and at the LC/ONH-level cause progressive loss of RGCs and their axons, resulting in thinning of the RNFL and reduced connectivity to the brain, which leads to visual impairment and can cause blindness unless treatment(s) are started for the patient (Figure 8).

## Treatment Options for Ocular Hypertension and Primary Open-Angle Glaucoma/Primary Closed-Angle Glaucoma/NTG

Several pioneering clinical trials in the early 2000s and animal models of OHT/glaucoma have provided compelling evidence that elevated IOP/OHT is a principle root cause of several forms of glaucoma. Consequently, a multipronged approach has been adopted to address this aspect of the disease. Thus, reducing IOP with ocular hypotensive drugs, removal of excess AQH from the ANC of the eye with microshunts and/or surgically promoting AQH drainage from the ANC of the eye of OHT/POAG/NTG patients slows down disease progression and preserve sight by reducing the death of RGCs and their axons (reviewed in Weinreb et al., 2014; Zhang et al., 2019).

### Pharmaceutical and Cellular Therapeutics to Lower Intraocular Pressure

**FIGURE 9:**
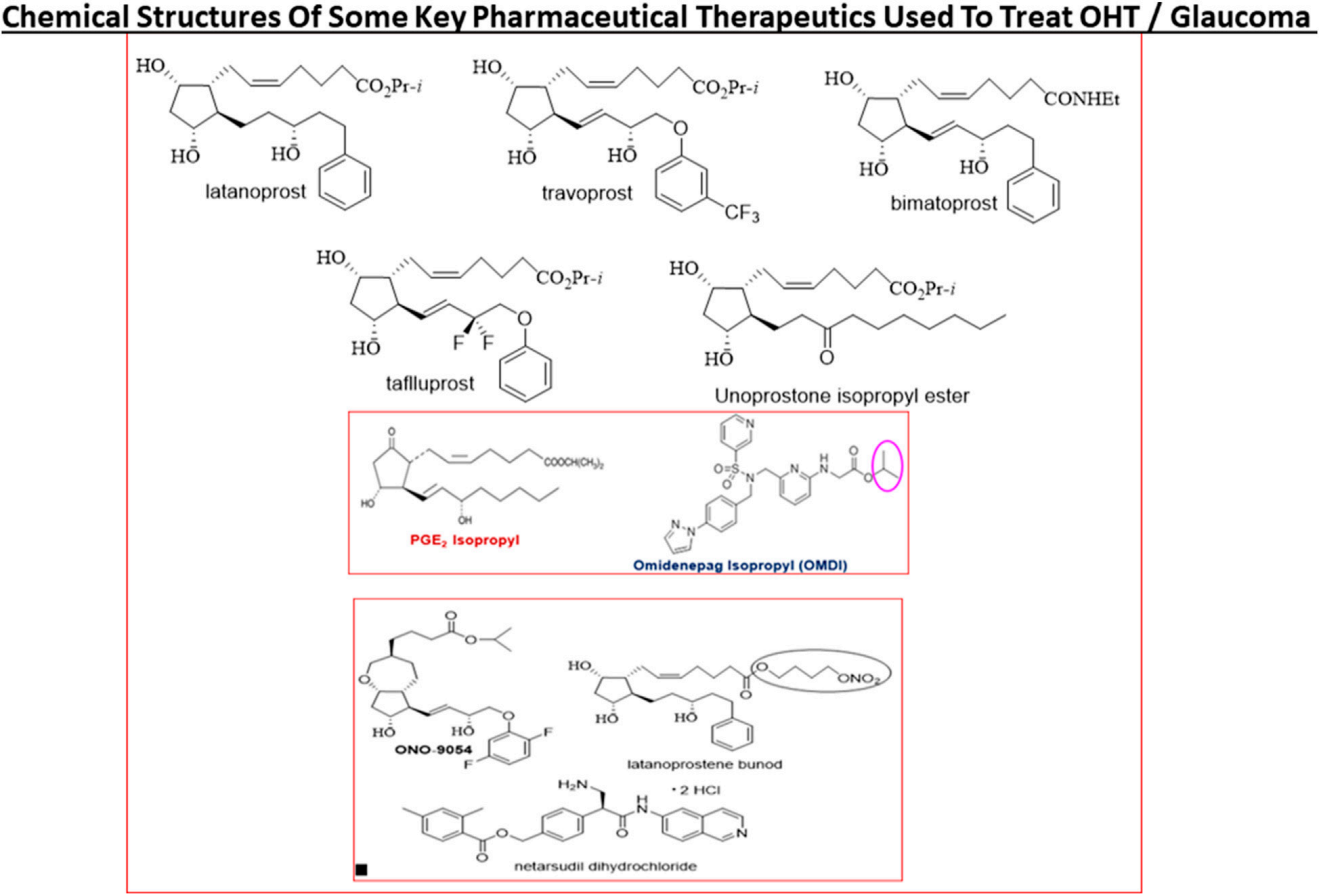
The chemical structures of some key pharmaceutical agents used to treat POAG are depicted. Many of the drugs have been approved by world health authorities for managing POAG.

The muscarinic receptor agonists acetyl choline and pilocarpine were the earliest therapeutical drugs used to lower IOP to treat glaucoma towards the end of the 19th century. Indeed, use of sympathetic and parasympathetic nervous system transmitters and new drug analogs continued for this purpose for a few decades including approval for adrenaline (1920), carbachol (1932), acecledine (1960), propanolol (1967), and clonidine (1972). However, many of these drugs were relatively non-selective for the family of receptors through which they imparted their ocular hypotensive activity. While more potent and slightly longer acting beta-adrenoceptor antagonists were soon discovered and approved for glaucoma treatment (e.g., timolol in 1978; carteolol in 1982, levobunolol in 1985), it was only thereafter that receptor selectivity began to be addressed. Thus, betaxolol (β-1-receptor selective antagonist approved in 1985; e.g., Sharif and Xu, 2004; Sharif et al., 2001) and apraclonidine (α2-receptor-selective agonist approved in 1987; brimonidine approved in 1997) were FDA-approved. New classess of ocular hypotensive drugs then emerged including carbonic anhydrase inhibitors (dorzolamide approved in 1994; brinzolamide approved in 1998) and FP-receptor-selective prostaglandin analog agonists including latanoprost (approved in 1996) and travoprost/bimatoprost (approved in 2001; see Hellberg et al., 2001; Hellberg et al., 2002; Sharif et al., 2002a; Sharif et al., 2002b) (Figure 9). Combination products were additionally approved to gain more efficacy including dorzolamide and timolol (approved in 1998) and brimonidine and timolol (approved in 2007). Newer drugs including a conjugate of latanoprost and an NO-donor (latanoprostene bunod, approved 2017; Cavet et al., 2014), two rho kinase inhibitors (ripasudil [approved in Japan 2014]; netarsudil [approved in US in 2017]) and a novel non-prostaglandin EP2-receptor-selective agonist (omidenepag isopropyl; approved in Japan 2018) are the most recently introduced drugs to lower and control IOP (Table 1; Duggan 2018; Kirihara et al., 2018a; Kirihara et al., 2018b; Fuwa et al., 2018; Fuwa et al., 2021; Ferro Desideri et al., 2019; Aihara et al., 2020a; Aihara et al., 2020b; Sharif et al., 2020) (Figure 9). Interestingly, some drugs like brimonidine and betaxolol, not only lower IOP but are also neuroprotective, at least in animal models of IOP-dependent and IOP-independent glaucomatous damage (e.g., Rusciano et al., 2017; Conti et al., 2021).

**TABLE 1:** Selected health authority-approved IOP-lowering agents and their properties.

Brand name of drug & year of clinical Introduction/FDA or EMEA or Japan approval (where known)	Generic name & drug type. (IOP reduction achieved in OHT/POAG patients)	Dosage type (%, w/v)	Topical ocular dosing frequency	Mechanism of action to lower IOP	Some Side-Effects/Adverse reactions
**Approved enhancers of conventional outflow (TM/SC pathway) of AQH to lower IOP**					
Isopto carpine (1974); Pilopine	Pilocarpine (muscarinic receptor agonist)	1, 2, 4%; 4% Gel	1 drop 2-4-times daily; single application of gel across the eye	Enhances conventional (TM) outflow of AQH	Brow-ache, miosis; accommodative change, eye irritation, eye pain, blurred vision, and/or visual impairment, potential tachycardia
Isopto Carbachol	Carbachol (muscarinic receptor agonist)	1.5, 3% solution	1–2 drops up to 3-times daily	Enhances conventional (TM) outflow of AQH	Brow-ache, miosis, accommodative change, eye irritation, eye pain, blurred vision, and/or visual impairment, potential tachycardia
Glanatec (2014 Japan)	Ripasudil (Rho kinase [ROCK] inhibitor) (3.5–4.5 mmHg IOP reduction)	0.4% solution		Enhances conventional (TM) outflow of AQH	Conjunctival hyperemia, allergic conjunctivitis, blepharitis, punctate keratitis
Rhopressa (2017)	Netarsudil (Rho kinase [ROCK] inhibitor) (5 mmHg IOP reduction)	0.02% solution	1 drop daily	Enhances conventional (TM) outflow of AQH; also decreases episcleral veinous pressure	Conjunctival hyperemia, corneal verticillata, instillation site pain, and conjunctival hemorrhage
**Approved Inhibitors of AQH Production to Lower IOP**					
Timoptic (1978) Timoptic-XE Gel	Timolol (beta-adrenoceptor antagonist)	0.25%, 0.5% solution or gel-forming solution	1 drop 1-2-times daily	Reduces production of AQH from CB	Signs and symptoms of ocular irritation, (e.g. burning, stinging, itching, tearing, redness), conjunctivitis, blepharitis, keratitis, dry eyes, decreased corneal sensitivity, blurred vision, corneal erosion. Visual disturbance, including refractive changes
Betoptic (1985)	Betaxolol (beta-1-selective adrenoceptor antagonist)	0.25% suspension; 0.5% solution	1 drop 2-times daily; 1–2 drops twice daily	Reduces production of AQH from CB	Transient ocular discomfort, Decreased corneal sensitivity, erythema, itching sensation, corneal punctate keratitis, anisocoria, blurred vision, foreign body sensation, tearing, dryness of eyes, inflammation, discharge, ocular pain, decreased visual acuity, crusty lashes and photophobia; Bradycardia, heart block; Pulmonary distress characterized by dyspnoea, bronchospasm, thickened bronchial secretions, asthma and respiratory failure; Insomnia, dizziness, vertigo, headaches, depression, lethargy
Alphagan (1996)	Brimonidine (2–6 mmHg IOP reduction)	0.15%, 0.2% solution	1 drop 3-times daily	Reduces production of AQH from CB and enhances UVS AQH outflow	Allergic conjunctivitis, conjunctival hyperemia, and eye pruritis; local ocular hypersensitivity; blurred vision, burning sensation of eyes, drowsiness, eye headache, stinging of eyes, foreign body sensation
Iopidine (1987)	Apraclonidine	0.5% solution	1–2 drops 3-times daily	Reduces production of AQH from CB	Hyperemia (redness), itching, tearing of the eye, Blurred vision or change in vision, chest pain, clumsiness or unsteadiness, depression, dizziness, eye discharge, irritation, or pain, irregular heartbeat
Trusopt (1994)	Dorzolamide (carbonic anhydrase inhibitor	2% solution	1 drop 3-times daily	Reduces AQH generation by the CB	Transient bitter taste and superficial punctate keratitis, eye irritation, burning, stinging, and ocular discomfort; blurred vision, excessive tearing, dry eyes, and increased sensitivity to light
Azopt (1998)	Brinzolamide (carbonic anhydrase inhibitor	1% suspension	1 drop 3-times daily	Reduces AQH generation by the CB	Temporary blurred vision, bitter/sour/unusual taste, dry eyes, temporary discomfort, itching, redness of the eye, foreign body sensation, eye discharge, and headache
**Approved Stimulators of UVS Outflow of AQH to Lower IOP**					
Xalatan (1996)	Latanoprost (FP-prostaglandin receptor-selective agonist	0.005% solution	1 drop at bedtime	Enhances AQH outflow via the UVS pathway and some via TM/SC pathway	Blurred vision, burning, stinging, itching, hyperemia, foreign body sensation, changes in eyelash number/color/length/thickness, iridial darkening, (pigmentation), periocular skin darkening, deepening of eyelid sulcus (loss of periorbital fat), dry eye, eyelid crusting and discomfort, increased sensitivity to light
Travatan (2001)	Travoprost (FP-prostaglandin receptor-selective agonist	0.004% solution	1 drop at bedtime	Enhances AQH outflow via the UVS pathway and some via TM/SC pathway	Blurred vision, burning, stinging, itching, hyperemia, foreign body sensation, changes in eyelash number/color/length/thickness, iridial darkening, (pigmentation), periocular skin darkening, deepening of eyelid sulcus (loss of periorbital fat), dry eye, eyelid crusting and discomfort, increased sensitivity to light
Lumigan (2001)	Bimatoprost (FP-prostaglandin receptor-selective agonist	0.03% solution	1 drop at bedtime	Enhances AQH outflow via the UVS pathway and some via TM/SC pathway	Increased conjunctival hyperemia, darkening of eyelids, increased thickening and number of eyelashes, dry eye, eye irritation, eye itching
					Hirsutism (a condition of hair growth on parts of the body normally without hair)
Taflotan (2008 Japan)	Tafluprost (FP-prostaglandin receptor-selective agonist	0.0015% solution	1 drop at bedtime	Enhances AQH outflow via the UVS pathway and some via TM/SC pathway	Ocular surface burning, stinging, irritation, hyperemia, foreign body sensation, dry eyes, watering eyes, iridial darkening, periocular skin darkening, abnormal eyelash growth, and increased sensitivity to light
Zioptan (2012 United States)					
Rescula (2000)	Unoprostone (FP-prostaglandin receptor agonist	0.15% solution	1 drop twice daily	Enhances AQH outflow via the UVS pathway and some via TM/SC pathway	Eye burning, stinging, dry eyes, itching, increased length of eyelashes, and injection; iridial darkening, blepharitis, cataract, conjunctivitis, corneal lesion, discharge from the eye, eye hemorrhage, eye pain, keratitis, irritation, and photophobia
Eybelis (2018 Japan)	Omidenepag Isopropyl (EP2-receptor selective non-prostaglandin agonist	0.002% solution	1 drop daily	Enhances AQH outflow via the UVS pathway and via TM/SC pathway	Transient conjunctival hyperemia, corneal thickening
**Some Approved Combination Products for Lowering IOP**					
Cosopt (1998)	Dorzolamide + timolol	2% + 0.5%	1 drop 2-times daily	Reduce AQH production from CB	Combination of side-effects from both drugs
Combigan (2007)	Brimonidine + timolol	0.2% + 0.5%	1 drop every 12 h	Reduce AQH production from CB	Combination of side-effects from both drugs
Simbrinza (2013)	Brinzolamide + Brimonidine	1% + 0.2%	1 drop 3-times daily	Reduce AQH production from CB	Combination of side-effects from both drugs
Roclatan (2019)	Netarsudil + Latanoprost	0.02% + 0.005%	1 drop daily	Enhancement of AQH outflow via TM/SC and UVS pathways	Combination of side-effects from both drugs
Xalacom	Latanoprost + timolol	0.005% + 0.5%	1 drop daily	Enhancement of AQH outflow and by inhibiting AQH production	Combination of side-effects from both drugs
Duotrav	Travoprost + timolol	0.004% + 0.5%	1 drop daily	Enhancement of AQH outflow and by inhibiting AQH production	Combination of side-effects from both drugs
Ganfort	Bimatoprost + timolol	0.03% + 0.5%	1 drop daily	Enhancement of AQH outflow and by inhibiting AQH production	Combination of side-effects from both drugs
Taflotan + timolol	Taflotan + timolol (>13 mmHg IOP reduction; 40% lowering)	0.0015% + 0.5%	1 drop daily	Enhancement of AQH outflow and by inhibiting AQH production	Combination of side-effects from both drugs
**Other Products for Lowering IOP**					
Vyzulta (2017)	Latanoprostene Bunod (conjugate of latanoprost and an NO-donor agent)	0.024% solution	1 drop at bedtime	Enhances AQH outflow via the UVS pathway and via TM/SC pathway	Eye discomfort/irritation, hyperemia, temporary blurred vision, increase in eyelash number/length/thickness and darkening of the eyelashes/eyelids and iris
Durysta Implant (2020)	Intracamerally injected sustained-delivery biodegradable polymer containing bimatoprost	Not applicable	Once implanted into the ANC of the eye (intracameral injection), the drug elutes off the implant over 6-months	Enhances AQH outflow via the UVS pathway and via TM/SC pathway	Conjunctival hyperemia, foreign body sensation, eye pain, photophobia, conjunctival hemorrhage dry eye, eye irritation increased IOP, corneal endothelial cell loss, vision blurred, iritis, headache

Today, first-line topical ocularly (t.o.) administered therapeutics utilized to lower and maintain IOP include several FP-receptor prostaglandin (PG) agonists such as latanoprost, travoprost, tafluprost and bimatoprost which all enhance UVSC outflow of AQH in POAG/NTG patients and are used once-daily (Table 1) (Sharif et al., 1999; Hellberg et al., 2001; Hellberg et al., 2002; Weinreb et al., 2014; Jonas et al., 2017; Sharif, 2017; Sharif, N. A. 2018; Sharif, 2020a). Recent evidence also suggests that the latter compounds also engage the conventional TM-based outflow of the AQH (Toris et al., 2008) by activating the FP-receptors located on the TM cells (Sharif et al., 2003b) to enhance TM/SC outflow. Percent IOP reductions from baseline are usually greater when the patient’s IOP is higher, and the PG analog agonist drugs provide 26–37% IOP lowering in OHT/POAG patients. For instance, travoprost 0.004% t. o. dosed once in the evening at bedtime yielded 7.1–8.4 mmHg IOP lowering from baseline at 8am-4pm the next day in OHT/POAG patients followed for up to 12 weeks (Hellberg et al., 2002) (Figure 9).

TM outflow promoting drugs include the muscarinic receptor agonist pilocarpine (Osterlin et al., 1994; Toris et al., 2001), rho kinase (ROCK) inhibitors such as Y-27632 (Honjo et al., 2001), ripasudil (Futakuchi et al., 2020) and netarsudil (Lin et al., 2018; Serle et al., 2018) and NO-producing drugs (e.g., latanoprostene bunod; Cavet et al., 2014). While pilocarpine and FP-receptor agonists lower IOP by promoting the release of MPPs from the TM/CM cells to help digest some of the ECM and thereby create holes for AQH to leave the ANC. Due to their lower efficacy, these drugs are t. o. dosed at least twice-daily (ROCK inhibitors) and ≥ 4-times-daily for pilocarpine. Ripasudil for instance lowered IOP in various forms of glaucoma by 19.4–23.4% from baseline followed over 1–6 months, while netarsudil yielded 23–24% reduction of IOP with OHT/POAG patients followed for 3 months (Futakuchi et al., 2020). The mechanism of action of the ROCK inhibitors is by way of relaxing the TM/CM and allow the larger surface area of the TM to efflux the AQH.

Last but not least, there are pharmaceutical agents that slow down the production of AQH in order to decrease IOP (see Table 1). Such AQH inflow inhibitor drugs (e.g., beta-blockers [timolol (e.g., 18.7–21.5% IOP-lowering]; betaxolol and levobetaxolol (Sharif et al., 2001; Sharif and Xu, 2004; Quaranta et al., 2007)), carbonic anhydrase inhibitors (brinzolamide; 17.8–18.5%), and alpha-2-adrenergic agonists (brimonidine; apraclonidine) are not very potent or highly efficacious but are effective enough to be used either alone (usually at least twice-daily) or in conjunction with other IOP-lowering drugs in fixed-dose combination products (Nakamura et al., 2009; Hollo et al., 2014; Tanihara et al., 2015; Lusthaus and Goldberg, 2017; Asrani et al., 2020). For example, fixed-dose tafluprost + timolol reduced IOP up to 40% (>13 mmHg) while netarsudil + latanoprost reduced IOP by 58.4%. Simbrinza (brinzolamide + brimonidine) is a novel and unique combination product that does not contain a beta-blocker and is best suited to treat patients that have pulmonary or cardiac issues. A recently approved ocular hypotensive drug, latanoprostene bunod (a conjugate of an FP-receptor agonist and a NO-donor) induces AQH outflow through both TM/SC and UVS pathways to decrease IOP (Cavet et al., 2014) (Figure 9).

Even though the health authority-approved ocular hypotensive drugs described above are effective at reducing IOP they all have various types of ocular, and in some cases, systemic side-effects (Table 1). Notably and for instance muscarinic agonists such as pilocarpine and carbachol cause browe ache, miosis, sweating, bronchospasm and diarrhea. Alpha-2-receptor agonists like brimonidine (Cantor, 2006) cause hyperemia, ocular allergy, contact dermatitis, apnea, hypotension and lose their IOP-lowering efficacy upon repeated dosing due to tachyphylaxis. Beta-blocker drugs like timolol cause side-effects like punctate keratitis, corneal anesthesia, bronchospasms, increased heart block, hypotension, and depression. Carbonic anhydrase inhibitors cause corneal/ocular surface irritation/hypermia, abdominal discomfort, aplastic anemia and slight weight loss. FP-prostgalndin agonists increase some hyperemia, iridial and periorbital skin darkening, deepening of the upper eyelid sulcus, eyelash thickening and growth in a multi-direction manner which is undesirable.

**FIGURE 10:**
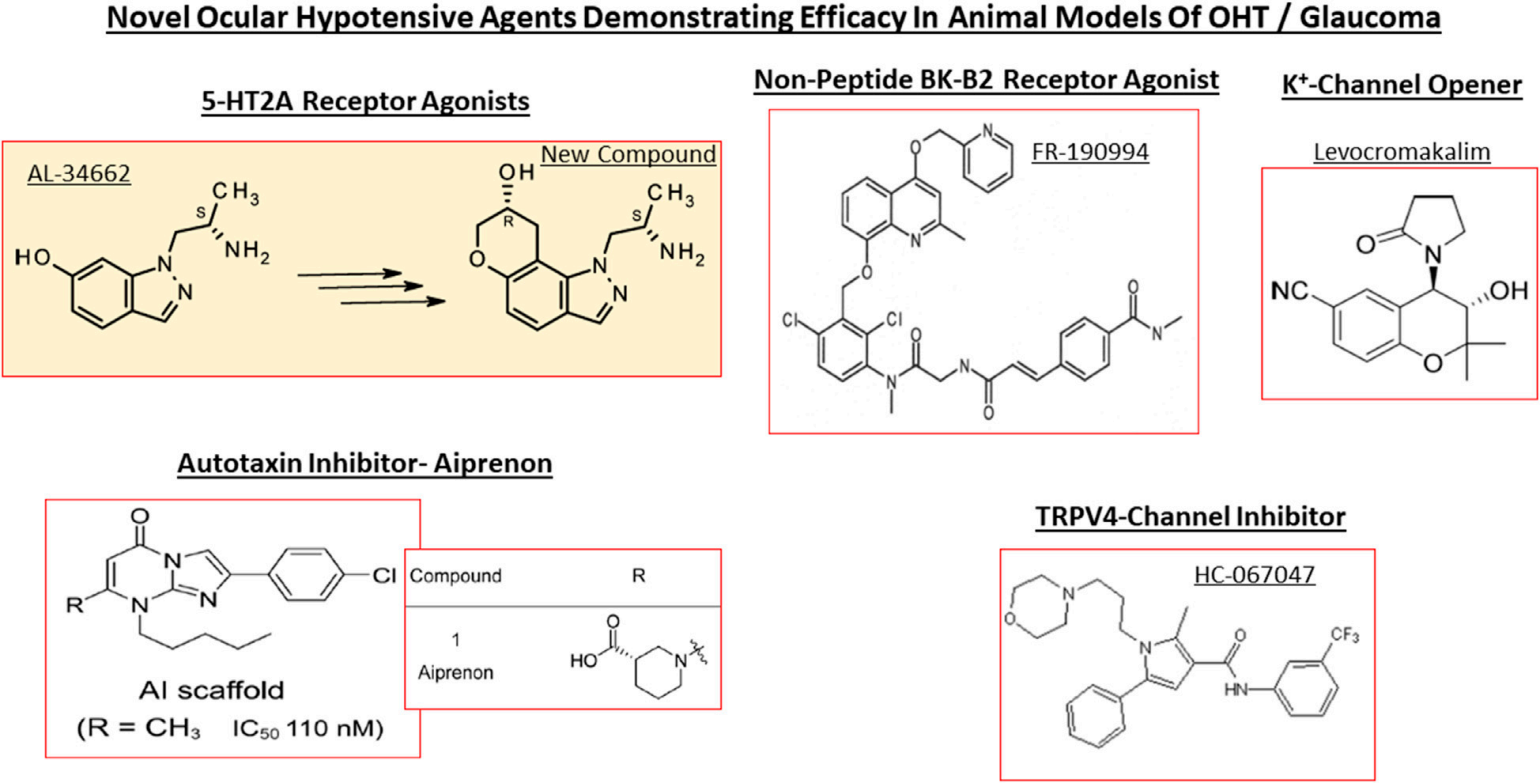
Chemical structures of some of the emerging ocular hypotensive agents are shown. The diversity of the chemical classes of compounds working through receptors, channels and enzymes is readily apparent.

Despite introduction of pharmaceutical/surgical and device treatment modalities into clinical management of OHT/glaucoma, more potent, more efficacious, more tolerable novel drugs that possess longer duration of action and/or have reduced side-effects and/or offer unique mechanisms of action are still being sought. Recent research has yielded new ocular hypotensive molecules of varying effectiveness based on animal models of OHT/glaucoma including using rodents, rabbits, dogs and monkeys (e.g., Table 2) (Weinreb et al., 2014; Donegan and Lieberman, 2016; Dikopf et al., 2017; Sharif, 2017; Sharif, 2018a; Sharif, 2018b). Some examples of new IOP-lowering therapeutics in early-stage research and development include the following: PG agonist possessing DP and EP2-receptor activities (e.g., AL-6598; Hellberg et al., 2002; Sharif et al., 2004); a dual agonist acting at FP and EP3 receptors (ONO-9054; Yamane et al., 2015); hydrogen sulfide donors (Salvi et al., 2016), melatonin receptor agonists (Alkozi et al., 2020), dopamine agonists (Bucolo et al., 2019), serotonin receptor-2A agonists (e.g., AL-34662 and cabergoline; Sharif et al., 2007; Sharif et al., 2009; Feng et al., 2007; May et al., 2015; Furlotti et al., 2018; Figure 10), adenosine A3 and other purinergic agonists (Galvao et al., 2015; Jacob et al., 2018), enantiomeric beta-blockers such as levobetaxolol (Sharif et al., 2001; Sharif and Xu, 2004; Quaranta et al., 2007), soluble guanylate cyclase activators (Dismuke et al., 2009), non-peptidic bradykinin receptor-2 agonists (Sharif et al., 2014a; Sharif et al., 2014b; Figure 10), constrained C-type natriuretic peptide mimetics (Savinainen et al., 2019), pigment epithelium-derived factor inhibitors (Rogers et al., 2013); K^+^-channel activators (Roy Chowdhury et al., 2017), autotaxin inhibitors (Honjo et al., 2018; Nagano et al., 2019; Nakamura et al., 2021; Figure 10), angiotensin (1–7) (Foureaux et al., 2013; Vaajanen et al., 2008), cannabinoid receptor agonists (Panahi et al., 2017), LIM kinase inhibitors (Goodwin et al., 2014), ligands for calcium voltage-gated channel auxiliary subunit alpha2delta 1 gene (Cacna2d1) target (Ibrahim et al., 2019), H_3_-histamine receptor antagonists (Lanzi et al., 2019), siRNA inhibitor against β-adrenoceptor (Gonzalez et al., 2014), melatonin (Martínez-Águila et al., 2016), digoxin-based Na^+^/K^+^-ATPase inhibitor drugs (Katz et al., 2016), K^+^-channel activators (Figure 10), TRPV4-channel inhibitors (Figure 10), aquaporin-1 inhibitors (Patil et al., 2016), and novel non-PG EP2-receptor agonists such as omidenepag isopropyl (Kirihara et al., 2018a; Kirihara et al., 2018b; Sharif et al., 2020), with latter actually having a dual mechanism of action and effectively lowering and controlling IOP in OHT/POAG patients (Aihara et al., 2020a). Next generation IOP-lowering drugs, that perhaps also possess neuroprotective activity, include FP-receptor PG-conjugates utilizing a number of different classess of ocular hypotensive drugs (Ellis D. et al., 2017). Recent glaucoma genetic studies (Aung and Khor, 2016; Aung et al., 2018; Choquet et al., 2020), and use of anti-sense oligonucleotide technology to suppress production of the damaging cytokine TGF-β2 (Fattal and Bochot, 2006; Pfeiffer et al., 2017) offer some hope of ameliorating pathologies of glaucoma. Other novel approaches to treat POAG involve using SC activators (e.g., AKB-9778) which inhibit vascular endothelial protein tyrosine phosphatase (VE-PTP) to activate tyrosine kinase with immunoglobulin-like and EGF-like domains 2 (Tie2) (Li et al., 2020); enhancers of autophagy such a rapamycin or tat-beclin (He et al., 2019; Zhu et al., 2020; Kasetti et al., 2021); delivering mitochondrial-targeted anti-oxidants (Chen et al., 2011; Rao et al., 2019); and by rejuvenating dying TM cells using stem cell-derived secretomes to lower and control IOP (Harrell et al., 2019). Additionally, perhaps exploitation of the newly discovered tunneling nanotubes to revive TM cells may offer some benefits in the future (Keller et al., 2017). Trace elements such a selenium, zinc, manganese and iron either alone or in combination with other drugs may also be useful in combating elevated IOP and glaucoma (Kamińska et al., 2021). Since the afore-mentioned compounds and technologies are at an early discovery stage, it is hoped that at least some of them will continue to be of clinical interest and will meet the necessary target product profile criteria to enter clinical trials in due course.

**TABLE 2:** Some examples of recently discovered IOP-lowering agents and their potential mechanisms of actions in various animal models of OHT.

Compound classes	Investigative agent	Reported or potential mechanism(s) of action (MOA)
Conventional Outflow (via TM pathway) Promotors		
Inhibitors of chloride transport	Ticrynafen; Ethacrynic acid; Indacrinone	Inhibition of Na^+^-K^+^-Cl^-^-transporter activity in the TM changes cell shape and volume and thus AQH efflux is increased
Kinase inhibitors	Chelerythrine; Staurosporin; LIM-K inhibitors (e.g., LX7101); Myosin-II ATPase inhibitor: Blebbistatin. Src kinase inhibitor	Modification of actomyosin contractility that leads to changes in actin cytoskeleton of TM and this leads to AQH efflux; direct relaxation of the TM may also be involved
Rho Kinase Inhibitors	Fasudil; Y-27632; AMA0076; ITRI-E-212	Modification of actomyosin contractility that leads to changes in actin cytoskeleton of TM and this leads to AQH efflux; direct relaxation of the TM may also be involved
Marine macrolids	Latrunculins A and B; Bumetanide; Swinholide	Promote sequestration of actin monomers and dimers in TM; cause cell TM shape change and thus AH efflux
Guanylate cyclase activators	Natriuretic peptides and constrained cyclic peptides: ANP; CNP; TAK-639	Type-A and type-B receptor activation leads to cGMP production, TM relaxation and AQH efflux via TM.
NO Donors	Sodium nitroprusside; Hydralazine; 3-morpholinosyndnonimine; (S)-nitrosoacetylpenicillamine; NCX-125	NO activates intracellular soluble guanylate cyclase to increase cGMP production, TM relaxation and AQH efflux via TM.
Soluble guanylate cyclase activators	IWP-953; MGV354	These compounds directly activate intracellular soluble guanylate cyclase to increase cGMP production, TM relaxation and AQH efflux via TM.
κ-opioid receptor agonists	Bremazocine; Dynorphin	Release natriuretic peptides and thus raise cGMP in TM leading to its relaxation and thus AQH efflux
Cannabinoid receptor agonists	WIN55212-2; CP55940; SR141716A	Receptor stimulation opens BKC-channels and relaxes TM which then causes AQH efflux via TM and SC
Serotonin-2 receptor antagonists	BVT-28949; Ketanserin and its analogs	Unknown and unverifiable mechanism(s) of action (may block beta-adrenergic receptors indirectly?)
Releasers of MMP & AP-1	*t*-butylhydroquinone (t-BHQ); β-naphthoflavone	Local production of MMPs; ECM degradation; stimulation of AQH efflux via TM/SC
Autotaxin/Lysophosphatidic acid inhibitors	Aiprenon	Promotion of AQH egress from TM/SC pathway
**Uveoslceral Outflow promotors (via CM bundles and sclera)**		
EP_2_- and EP_4_- PG-receptor agonists	AL-6598; Butaprost; ONO-AE1-259–01; PF-04217329; PF-04475270	Receptor activation increases cAMP that relaxes CM & TM; EP_2_ agonists also cause release of MMPs that breakdown ECM (“clog”) around CM bundles and within sclera thus causing UVS outflow of AQH
Serotonin-2 (5HT-2) receptor agonists	(R)-DOI; α-methyl-5HT; AL-34662	Contraction/relaxation of CM and TM by activation of 5HT_2_ receptors. May also release MMPs and/or PGs or other local mediators that promote CM remodeling and thus promote UVS outflow
Bradykinin B_2_-receptor agonists	Bradykinin; FR-190997; BKA278	B_2_-receptor activation causes PI hydrolysis production of IPs and DAG; cause PG release and release of MMPs that digest ECM and this promote UVS outflow in cynomolgus monkey; conventional outflow also stimulated in isolated bovine/porcine anterior eye segments ^[177,178]^
Dual pharmacophore PGs	FP/EP3 receptor agonist (ONO-954)	Promote UVSC outflow
Inflow inhibitors (reduce AQH production)		
Chloride channels inhibitors	5-nitro-2-(3-phenylpropylamino)-benzoate (NPPB)	Ion flux of CP NPE cells causes reduction of AQH formation
Na^+^-K^+^-ATPase inhibitors	Ouabain; Digoxin analogs	Ciliary process Na^+^-K^+^-ATPase inhibited leading to inhibition of AQH production
Dopamine receptor agonists	PD128907; CHF1035; CHF1024; SDZ GLC-756; (S)-(-)-3-hydroxyphenyl)-N-n-propylpiperidine (3-PPP)	Inhibit release of NE & prevent AQH production; may also release natriuretic peptides
Na^+^-K^+^-ATPase inhibitors	Ouabain; Digoxin analogs	Ciliary process Na^+^-K^+^-ATPase inhibited leading to inhibition of AQH production
Aquaporin Inhibitors	Various aromatic sulfonamides and dihydrobenzofurans	Inhibit release of NE & prevent AQH production
**Other IOP-lowering agents**		
Mas receptor stimulator	DIZE via ACE-2 activation	Prevent ECM (including TGFβ) accumulation (outflow stimulation ?)
Angiotensin-II receptor antagonists	CS-088	Various mechanisms of action; not robust IOP-lowering
Ca^2+^-channel inhibitors	Lomerazine; Nivaldipine; nifedipine; Nimodipine; Verapamil; Brovincamine; Iganidipine	Enhance retinal blood flow; some may lower IOP; work well in normal tension glaucoma patients
Alpha-adrenergic receptor antagonists	Oxymetazoline; 5-methylurapidil; Ketanserin	Work mostly via outflow mechanism but this needs to be defined
ATP-sensitive K^+^-channel activators	Cromakalim; Levocromakalim; CKPL1	Purported MOA involving episcleral veinous pressure modulation

Over the last 4-decades it has become evident that due to the various side-effects mentioned above, and due to the fact that most of the glaucoma patients are elderly, routine administration of their eyedrops is either forgotten, or is reduced or purposely missed (Newman-Casey et al., 2015). Mitigation efforts dealing with compliance isssues has resulted in development of several different technologies to safely and durably help deliver the IOP-lowering drugs to the patients target eye tissues (Shouchane-Blum et al., 2019). Some of the technologies include sustained release of IOP-lowering drugs from liposomes and nanoparticles cross-linked collagen shields (Monem et al., 2000; Agban et al., 2016) or just nanocarrier-based formulations (Natarajan et al., 2014; Wong et al., 2014), and via gel/microspheres (Fedorchak et al., 2017), from punctal plugs (Perera et al., 2016), via biodegradable polymers intracamerally injected into the ANC (Navratil et al., 2014; Lee et al., 2019) (Table 1), elution from contact lenses (Ciolino et al., 2016), from intravitreal injections of polycaprolactone drug delivery implant (Kim et al., 2018), and from silicone rings that are placed around the eyeball (Brandt et al., 2016; Lewis et al., 2017).

In terms of novel techniques and tools to better lower and control IOP involve the following experimental regimen: use of AAV-vectors delivering genes to TM and/or corneal endothelial cells and/or using aptmers to initiate local production/release of MMPs into the TM to digest collagen and other occluding proteins to promote AQH outflow hold great promise (Borrás, 2017; O’Callaghan et al., 2017; Fattal et al., 2018); CRISPR-Cas9-based treatment against misfolded myocilin in the ANC (Jain et al., 2017) and its use to delete aquaporin-1 in the CB (Wu et al., 2020); the potential delivery and intercollation of new TM cells induced from induced pluripotent stem cells into the existing TM structure to enhance the local phagocytic capability and capacity may be possible with some potential success (Ding et al., 2014; Sun et al., 2017; Zhu et al., 2016, 2017); the nonsurgical reduction of IOP after suprachoroidal injection of hyaluronic acid hydrogel for IOP control (Chae et al., 2020), and epigenetically rejuvenating the ANC cells of the TM and SC (Lu et al., 2020). Additionally, antioxidants can be used to enhance viability of TM cells in the ANC (Ammar et al., 2012), especially since oxidative stress (Rao and Stubbs, 2021) damages the DNA of the TM cells of POAG patients (Saccà et al., 2005; Abu-Amero et al., 2006). Senolytic drugs are also useful including quercetin, venetoclax, imatinib (El-Nimri et al., 2020).

The strong correlation between elevated IOP in the ANC of the eye, the IOP spikes, and glaucoma, and the reduction of GON damage by IOP reduction has been clearly demonstrated clinically in OHT/POAG patients (CNTGSG, 1998; AGIS Investigators, 2000; Heijl et al., 2002; Kass et al., 2002; Gordon et al., 2002; Leske et al., 2007; Medeiros et al., 2008; Musch et al., 2011; Figure 7). The current pharmacotherapies aim to decrease IOP by 20–50% and several classess of drugs are available for managing and reducing the progression of glaucoma, in particluar POAG, by enhancing AQH outflow and by inhibiting its formation. Due to the side-effect profiles of existing ocular hypotensive therapies discussed above, new and improved drugs to treat glaucoma are still needed.

### Laser-Treatments to Reduce Intraocular Pressure

Unfortunately for those OHT and POAG patients whose elevated IOPs remain uncontrolled despite maximal adjunctive eyedrop-based pharmaceutical therapy, laser- or surgical-based treatment needs to be deployed (Conlon et al., 2017; Geffen et al., 2017; Pahlitzsch et al., 2017; Garg and Gazzard, 2018; Lusthaus and Goldberg, 2019). For treating PACG for instance, surgical iridectomy (removing a part of the iris) has been replaced by Nd:YAG laser iridotomy, because it greatly reduces the possibility of suprachoroidal hemorrhage occurance. Laser peripheral iridotomy is used to quickly make tiny holes in the iris to help AQH to escape from the ANC in order to lower IOP. Likewise, selective laser trabeculoplasty (SLT) to open up the TM is a very helpful and safe procedure (Garg and Gazzard, 2018; Lusthaus and Goldberg, 2019). In SLT, a Nd:YAG laser is used to selectively target melanocytes and other cellular debris in the TM and thus less damage is exerted on the TM cells themselves. The exact mechanism by which SLT functions is not completely understood but mild inflammation created by the laser appears to release MMPs from the surrounding tissues which help clear the filtration system and promote AQH outflow from the ANC and thus lower IOP. Unfortunately, the latter technique often has to be repeated to lower and maintain the IOP below a threshold needed to preserve vision.

### Surgical Procedures to Lower Intraocular Pressure

**FIGURE 11:**
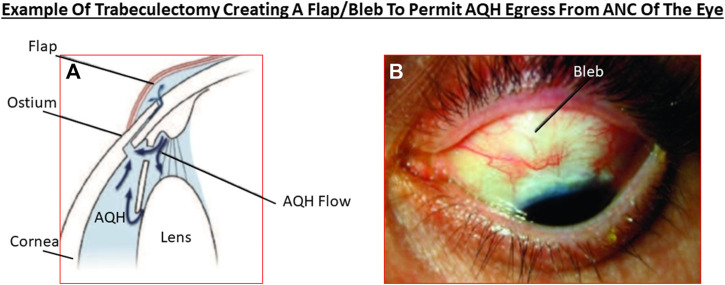
The technique of trabeculectomy to allow the excess AQH to leave the ANC to reduce IOP is shown in relation to other ANC ocular tissues **(A)**, and how a patient’s eye looks after the surgery and the position of the bleb/flap **(B)**.

One of the most well known and utilized surgical procedure to lower IOP is so-called filration surgery (Pahlitzsch et al., 2017; Conlon et al., 2017; Lusthaus and Goldberg, 2019). Here, a surgical anterior sclerotomy is conducted to create an AQH drainage pathway from the ANC to the ocular surface (usually under the conjunctiva) via a “bleb” to permit the lowering of IOP. Trabeculectomy involves removal of a portion of the TM to help remove AQH from the ANC. Bleb-forming procedures include ab externo trabeculectomy, cutting from the exterior of the eye to reach SC, TM and the ANC (Pahlitzsch et al., 2017; Conlon et al., 2017; Lusthaus and Goldberg, 2019) (Figure 11). Non-penetrating trabeculectomy is an ab externo (from the exterior) surgical process expose the SC via a large and deep scleral flap. The inner wall of SC is stripped off after surgically exposing the canal. Deep sclerectomy is a filtering surgery where the internal wall of SC is excised allowing subconjunctival filtration without actually entering the ANC. In order to prevent wound adhesion after deep scleral excision and to maintain good filtering results, it is sometimes performed with a variety of biocompatible spacers or devices, such as a collagen wick and collagen matrix. Viscocanalostomy involves injection of a viscoelastic substance into the SC in order to open up the canal and the AQH collector channels to help drain the AQH from the ANC to lower IOP (Conlon et al., 2017; Pahlitzsch et al., 2017; Lusthaus and Goldberg, 2019). Surgical/laser goniotomy involves severing the fibers of the TM to permit AQH to drain more freely from the ANC. A cyclogoniotomy is a surgical procedure for producing a cyclodialysis, in which the ciliary body is cut from its attachment at the scleral spur under gonioscopic control in order to reduce the production of AQH.

Canaloplasty involves making an incision into the eye to gain access to SC similar to viscocanalostomy. Here, a microcatheter is inserted into the canal around the iris to enlarge the main and smaller collector drainage channels. Upon removal of the catheter a suture is placed within the canal and tightened in order to open the SC and thus reduce the resistance and enhance outflow of AQH. This technique is much safer than traditional trabeculectomy since a bleb is not created and risk of infection and hypotony is eliminated (Conlon et al., 2017; Pahlitzsch et al., 2017; Lusthaus and Goldberg, 2019). Canaloplasty combined with cataract surgery is useful in gaining additive lowering of IOP in patients where both procedures are possible. Other surgical procedures for enhancing AQH dynamics to lower IOP include: trabeculopuncture using a Nd:YAG laser to punch small holes in the TM; goniocurretage process which permits removal of damaged or pathological TM tissue from inside the ANC; cyclodialysis, which separates the CB from the sclera to form a conduit between the suprachoroidal space and the ANC. Lastly, procedures that decrease production of AQH include destruction of some of the ciliary epithelial cells of the CB by cyclocryotherapy (using a freezing probe) or via cyclophotocoagulation using a transscleral laser procedure (Conlon et al., 2017; Pahlitzsch et al., 2017; Lusthaus and Goldberg, 2019).

### Minimally Invasive Glaucoma Surgeries-Based Devices for Lowering Intraocular Pressure

**FIGURE 12:**
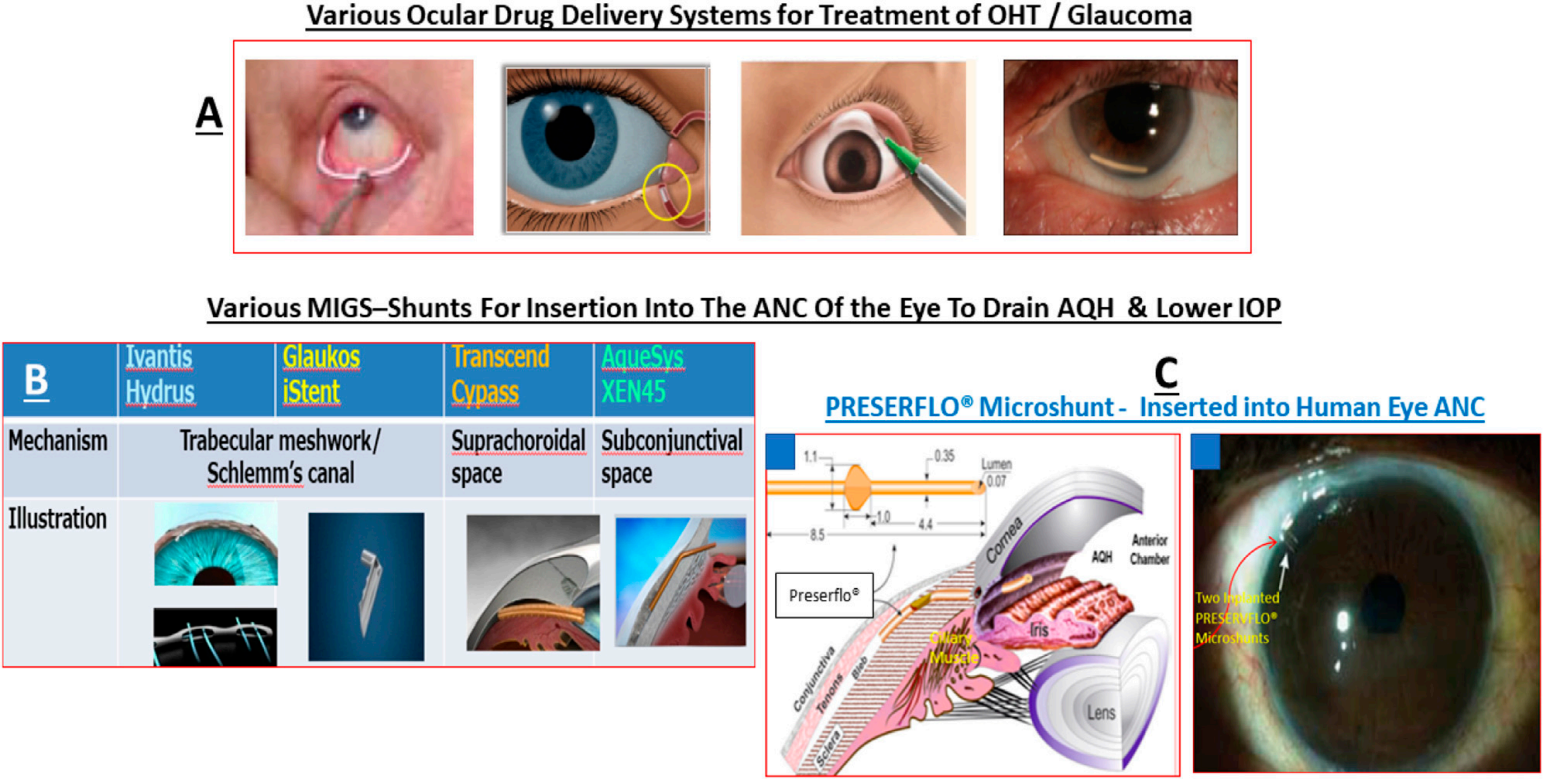
This montage depicts the various sustained ocular hypotensive drug delivery techniques/technologies **(A)**, and the various MIGS (AQH shunts) available to remove excess AQH from the ANC of the eye to reduce IOP **(B,C)**.

As described above, moderate-to-severe and refractory OHT/glaucoma can be treated using trabeculectomy and tube shunt surgery procedures, the most commonly utilized surgical techniques to-date. However, these incisional surgeries have significant safety and other issues and require significant post-operative medical care, including a continued high dependence on t. o. medications and the fact that these operations have to be frequently repeated to keep the outflow pathways opened. Micro-invasive glaucoma surgeries (MIGS; Ansari, 2017; Pillunat et al., 2017; Pahlitzsch et al., 2017; Ittoop et al., 2019; Nichani et al., 2020), or minimally invasive glaucoma surgery (Figure 12), represent a novel platform of surgical techniques and implants that overcome many of the latter issues and are highly effective in reducing and controlling IOP with a significantly reduced dependance on t. o. ocular hypotensive drugs. MIGS lower and control IOP by either increasing AQH outflow by circumventing the TM, or via accessing the subconjunctival AQH drainage pathway, or enhancing UVS-outflow through the suprachoroidal spaces (Conlon et al., 2017).

**FIGURE 13:**
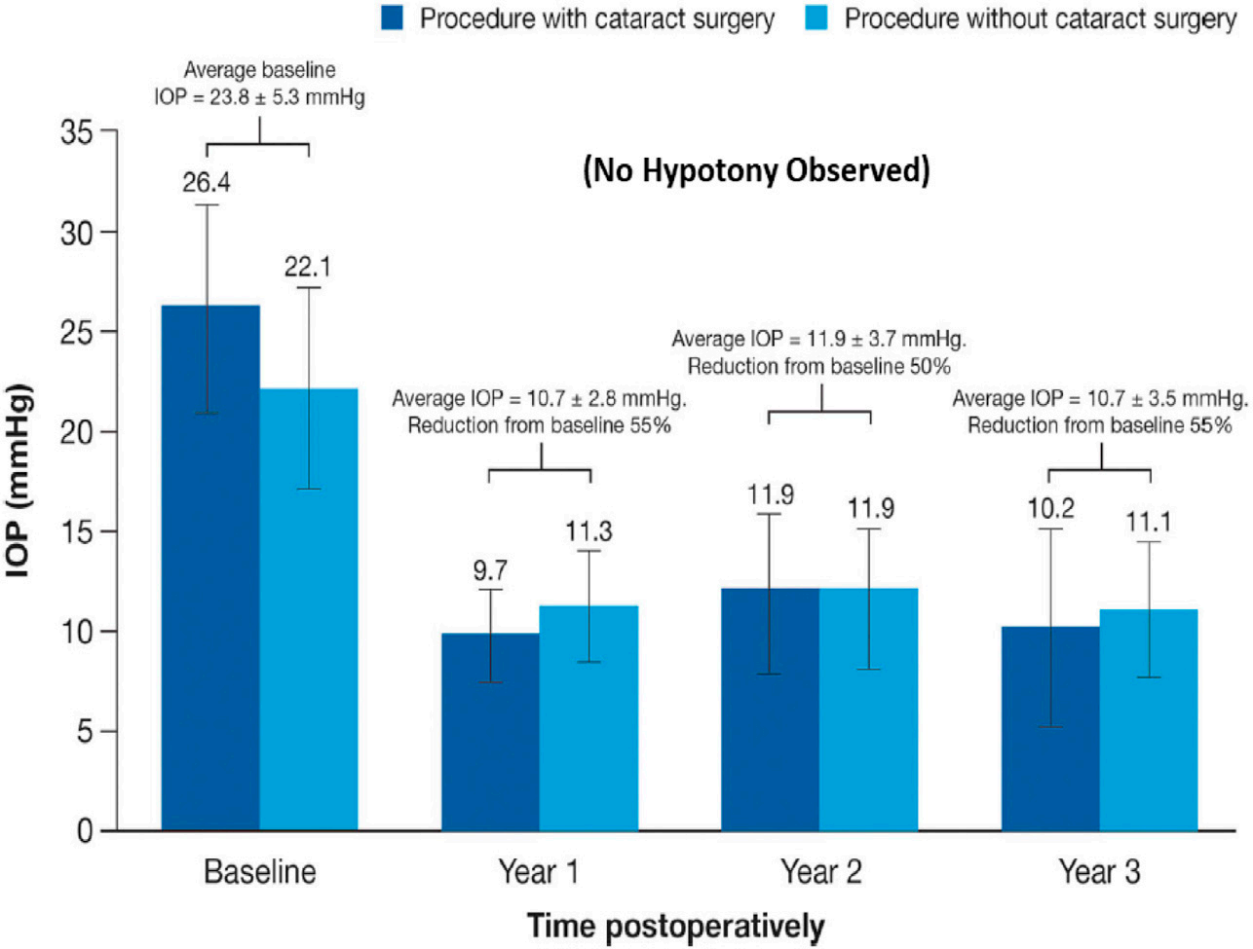
IOP reduction over 3 years in OHT/POAG patients after implantation of the PRESERFLO^®^ microshunt into the ANC to permit AQH egress to lower IOP is depicted.

**FIGURE 14:**
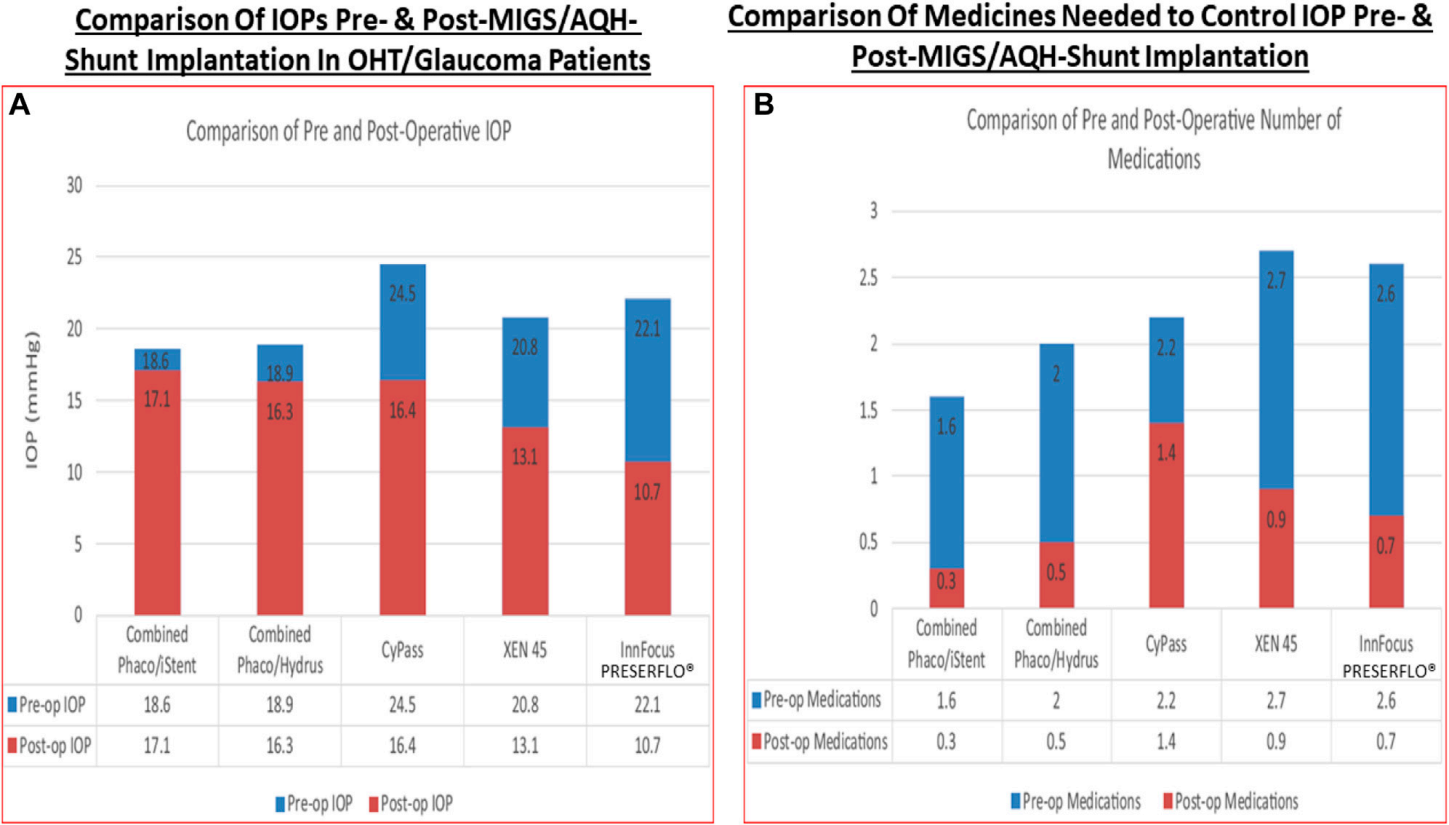
A comparison between various AQH microshunts on their ability to lower IOP in OHT/POAG patients **(A)**, and the need for number of ocular hypotensive medications needed to maintain the lowered IOP pre and post-MIG implantation **(B)** is shown.

Several MIGS procedures and associated AQH-shunt implants have entered into clinical management of OHT/glaucoma but they differ in terms of the technique (ab interno *vs* ab externo) utilized to insert the device and the type of microshunt that is utilized to drain the AQH from the ANC to lower IOP. Additionally, the target patient population, whether a bleb is used, the overall IOP-reduction achieved, ocular advere-events observed, and the relative need for t. o. drugs post-operation to maintain IOP help differentiate between the various MIGS available today. Since only modest reductions in IOP have been achieved with most MIGS procedures thus far, there remained an unmet medical need for a MIGS treatment to deal with moderate-to-severe and refractory glaucoma (Conlon et al., 2017). Therefore, the invention of a synthetic, elastomeric biomaterial (poly [styrene-block-isobutyleneblock-styrene]; SIBS) that is thermoplastic and resists biodegradation, helped in the development of a novel and unique SIBS-based AQH drainage device known as the PRESERFLO^®^ MicroShunt (formerly termed the InnFocus MicroShunt) (Sadruddin et al., 2019). This MicroShunt is an ab externo subconjunctival glaucoma AQH drainage device that facilitates AQH outflow to a bleb where the fluid is reabsorbed and sent into the veinous circulation thereby providing substantial IOP reduction. The average clinically useful % reductions in IOP (50–55%) achieved and maintained IOPs in the range of 10.7–11.9 mmHg over 3-years have been reported for PRESERFLO^®^ Microshunt (Figures 12–14). This MIGS-based implant device has therefore demonstrated a high efficacy (without hypotony) in enhancing AQh efflux from the ANC and thereby lowering and controlling IOP in reducing OHT/POAG patients (Sadruddin et al., 2019) (Figures 12–14).

## Drug Discovery Research for Ocular Hypertension/Intraocular Pressure-Lowering Drugs

In order to provide a perspective of the drug discovery processes involved in finding suitable ocular hypotensive drugs a brief outline of the *in vitro* assays and *in vivo* animal models used in such endeavors will be provided next.

### Assays and Techniques Used for Screening of Potential Ocular Hypotensive Compounds

Majority of the health authority approved drugs to treat a multitude of diseases that beset animals and humans exert their effects by modulating activity of cellular targets such as enzymes, receptors, ion-channels and transporters. Organic compounds, both naturally occuring (e.g., cyclosporin for dry eye; Daull et al., 2021) and synthetic drugs (e.g., olopatadine for ocular allergies (Sharif, 2020a,b); travoprost/tafluprost (Sharif et al., 1999; Hellberg et al., 2001, 2002) and omidenepag isopropyl ester (Kirihara et al., 2018a; Aihara et al., 2020a) to treat OHT/glaucoma), peptides (e.g., angiotensin-II and bradykinin; Sharif, 2015), antibodies (avastin; ranibizumab; aflibercept; Asahi et al., 2021) and most recently gene-therapy-based (aptamers; Fattal and Bochot, 2006; Khatib and Martin, 2017) medicines (and potential future drugs) have been discovered, characterized and introduced into medical treatment of diseases of the eye.

**FIGURE 15:**
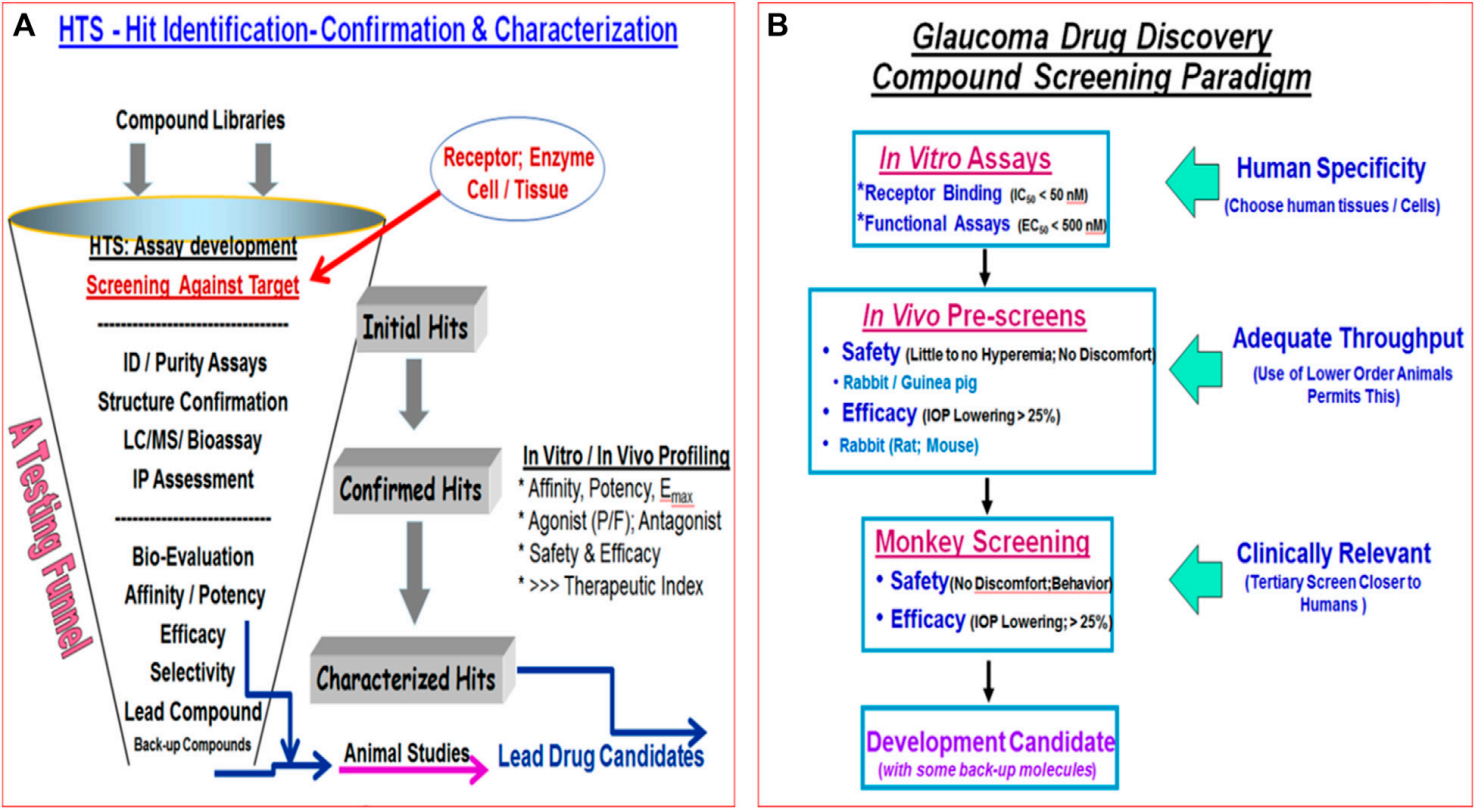
Examples of testing funnels for screening potential ocular hypotensive drug candidates are shown **(A,B)**. In (A), the overall “hits” discovery and characterization is depicted, whereas in (B), the types of stage-gates and the Go/No Go criteria to progress for selected *in vitro* and *in vivo* studies are shown. Potency/efficacy/safety parameters are listed as examples.

**FIGURE 16:**
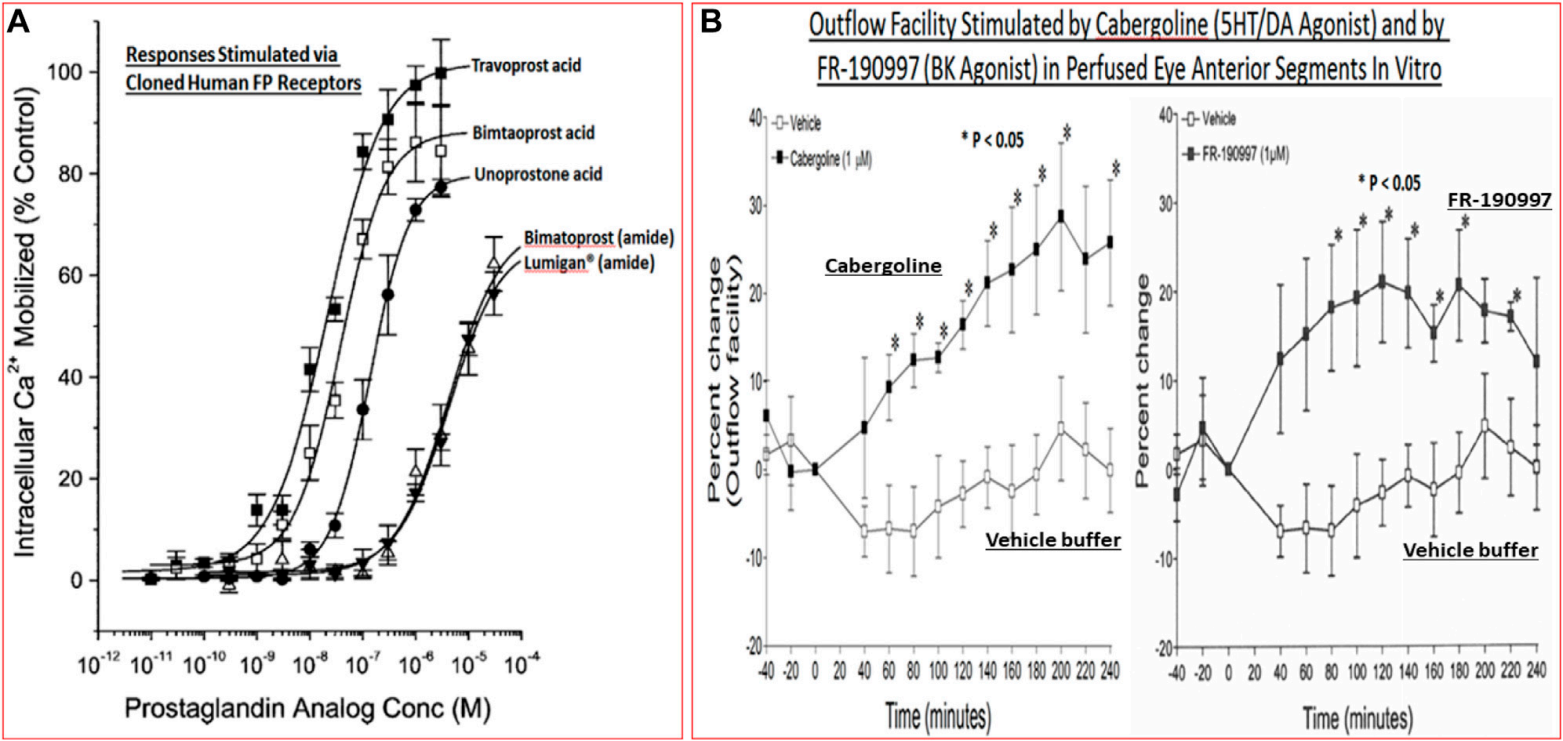
Examples of *in vitro* cell-based functional data for selected prostaglandins for their concentration-dependent mobilization of intracellular Ca^2+^ via the human cloned FP-receptor is shown **(A)**. *Ex-vivo* data for two different classes of compounds enhancing outflow facility in porcine eye ANC segments over a period of time is displayed **(B)**.

From an anti-glaucoma drug discovery perspective, the most important task for the investigator(s) is the identification of a suitable target to pursue that addresses an unmet medical need as described or defined by key opinion leaders, clinicians and patients of the OHT/POAG field. The target concept profile (TCP) or target product profile (TPP) then needs to be defined based on clear, objective, achieveable and desired attributes of the final drug candidate. Some of this information may exist in the literature or may be entirely proprietary. However, the knowledge of the target protein and disease mechanism and/or pathway is important in order to render the target into a compound screening funnel. In order to ensure that the target mRNA/protein is located in the normal and diseased target cells/tissues needs to be ascertained using RT-PCR (Sharif and Senchyna, 2006), or *in situ* hybridization (Ma et al., 1996), or receptor autoradiography (Sharif et al., 1999; Sharif et al., 2001; Davis and Sharif, 1999) and/or immunohistochemical (Sharif et al., 2014a; Sharif et al., 2014b) techniques. This is often followed by establishing suitable *in vitro* ligand-protein and/or protein-protein interaction assays to determine the relative affinity of test agents for the target protein (receptor/enzyme; e.g., Sharif and Xu, 1999; Sharif and Xu, 2004). Since it is important to determine whether the test compounds achieve appropriate engagement of the target receptor/enzyme, functional cell/tissue-based assays need to be employed. Human and/or non-human primate ocular cells and tissues, or cells expressing human cloned receptors or enzymes, should be used whenever possible, to have appropriate linkage to the human disease (Sharif N. A. 2018; Sharif, 2020a; Sharif, 2020b). Ideally, isolated and well characterized human CM (Sharif et al., 2003a), TM (Sharif et al., 2003b; Sharif et al., 2006) and NPCE (Crider and Sharif, 2001) cells, or bovine TM cells (Mao et al., 2012), or strips of human/animal ocular tissues (Wiederholt et al., 2000; Rosenthal et al., 2005; Ohia et al., 2018; Njie-Mbye et al., 2018) or *ex-vivo* models (e.g., Sharif et al., 2009; Sharif et al., 2014b) should be used for testing and characterizing ocular hypotensive compounds. If this is not possible, then suitable higher order species-derived cells and tissues may suffice including those from bovine, porcine and non-human primate sources. Such functional assays yield the relative potency and intrinsic activity of the test compounds in order to permit rank ordering of the agonist compounds. Obviously, if antagonists are being sought then a positive control, natural agonist ligand for the target would be employed to stimulate a response against which the potential antagonist/inhibitor compounds are screened. In order for the test compounds to pass the first series of stage-gate criteria, such testing funnel *in vitro* assays should require compounds to meet or exceed stringent affinity/potency/intrinsic activity, parameters defined at the onset of the screening campaign (Figures 15A,B). The initial hits should then be subjected to further scrutiny using additional *in vitro* primary and secondary assays (on-target and off-target), including testing in receptor subtype-based/enzyme isoform-based assays in order to determine relative selectivity of the compounds for the desired target protein (sub-type or isoform). Thus for example, for 5-hydroxy tryptamine-2 (5-HT2; serotonin-2) receptor agonist IOP-lowering compounds, not only was the affinity, agonist potency and intrinsic activity required at each of 5-HT2 receptor sub-types (5-HT2A, 5-HT2B, and 5-HT2C) but also at each of the 6 other major 5-HT receptor classes (i.e., 5-HT1 - 5-HT-7), and if possible at their subtypes (May et al., 2003; Sharif and Senchyna, 2006). A similar situation prevailed for various functionally and pharmacologically defined prostaglandin receptors and their sub-types which required establishment and validation of (Sharif et al., 2003). Minimal *in vitro* compound safety/toxicity and stability information, in a prototypic formulation, can also be gathered for the initial lead compounds in order to help select compounds for the next phase of screening. Functional read-outs from cultured cells in multi-well plates used to determine such quantifiable activity are second messengers (e.g., cAMP (Crider et al., 1998; Crider et al., 2000)/cGMP (Dismuke et al., 2009; Katoli et al., 2010)); inositol phosphates accumulation (Sharif and Xu, 1999; Griffin et al., 1997; Griffin et al., 1998; Sharif et al., 1998)/intracellular Ca^2+^ ([Ca^2+^]_i_) mobilization (Griffin et al., 1997; Griffin et al., 1998; Kelly et al., 2003) (Figure 16A), or enzyme release and activation (Sharif et al., 2003; Sharif et al., 2014a; Sharif et al., 2016), or cell contraction/relaxation by in-gel assays (Ramachandran et al., 2011) or by electrical resistance measurements (Park et al., 2015; Vollmer et al., 2020), or cell volume change (Patil et al., 2016; Dismuke et al., 2009; Dismuke et al., 2010). Tissue samples mounted in organ-baths (e.g., iris sphincter muscle; Sharif et al., 2008), rat uterus strips (Sharif, 2008) or bovine ciliary muscle/arteries (Njie-Mbye et al., 2018; Ohia et al., 2018) or human TM strips (Wiederholt et al., 2000) can provide functional read-outs of contraction or relaxation which can be quantified and test compounds rank ordered according to their relative potencies and intrinsic activities/maximal efficacies. For example, travoprost acid (TA) most potently contracted cat iris strips compared to all the other FP-receptor PG agonists tested, and TA was also a full agonist relative to the other compounds tested which clearly behaved as partial agonists in this assay system (Sharif et al., 2008). However, it is important to use multiple assay systems (if possible, in parallel to save time) and cross-correlate the findings to human ocular cells where possible in order to add value and to confirm the findings across species and test platforms. If the compound(s) meet the desired Go-criteria at this stage-gate, they can then be progressed to *ex-vivo* and *in vivo* testing paradigms. It is worth remembering that the afore-mentioned testing funnel-derived data are shared with the medicinal chemists on a regular basis such that the structure-activity information can be utilized to improve the next series of compounds that are designed and synthesized.

### *Ex-Vivo* Screening Models to Evaluate Potential Intraocular Pressure-Lowering Agents

Next, compounds satisfying the selection criteria from *in vitro* assays need to be evaluated for their ocular hypotensive activity. They can be first tested in bench-based *ex vivo* models using perfused ANC segments of eyes of suitable animals or postmortem human eyes. Since the CB and TM are retained and are functional, data obtained from such studies can yield valuable information about the ability of test perfused compounds to influence the outflow facility. Other mechanistic aspects such as the potential involvement of released substances/proteins (e.g., MMPs) can also be ascertained by sampling the effluent from these perfused segments. Such data has been reported using partially intact *ex-vivo* ANC eye segments of numerous different species (Llobet et al., 1999; Crosson et al., 2005; Webb et al., 2006; Keller et al., 2017; Mao et al., 2012; Boussommier-Calleja et al., 2012; Ren et al., 2016; Snider et al., 2021), and also using a whole perfused eye models (Shahidulla et al., 2005; Zhou et al., 2017). Figure 16B depicts example data obtained for two IOP-lowering agents, cabergoline and FR-190997, which enhanced outflow of perfused media in a time-dependent manner from such *ex-vivo* models.

### *In Vivo* Animal Models of Ocular Hypertension/Glaucoma Used for Screening Compounds

Testing of compounds for ocular safety and efficacy requires different stage-gates within the screening funnel. It is important to first ensure that the t. o. dosed test compounds dissolved or suspended in a comfortable formulation do not cause unacceptable ocular irritation, pain or other damage to the ocular surface. Typically, rodents, guinea pigs and rabbits are utilized for such ocular comfort studies using either a single high t. o. dose or repeated dosing at an intermediate concentration of the test drug. Preferably such tests should be performed in one of the eyes, and ideally, the test substances should not cause excessive eye redness (hyperemia), excessive blinking, tearing and conjunctival discharge, swelling of the eye lids, and vocalization in single or multi-dose topical ocular studies. The vehicle alone should be used as a control in the contralateral eye. When compounds pass the acute/chronic ocular safety process, and general systemic/central nervous system (CNS) safety (e.g., lack of effect on respiration, heart rate, CNS-related behaviors, etc), they can then be tested for their ability to lower IOP. If test agents are suspected of having a topical ocular anesthetic effect, they can be tested for such activity, and/or irritability potential, using electrophysiological recordings of corneal nerves but often these tests are performed after reproducible IOP-lowering effects have been determined (e.g., Sharif et al., 2014b).

Following successful passing through the ocular irritation stage-gate, compounds can now be tested for their ability to lower IOP. The test agents are again prepared in the selected formulation and are dosed t. o. at a single or multiple concentrations/doses to determine their relative efficacies to reduce IOP. Since the non-human primates are the most precious, costly and more labor-intensive to train and utilize for ocular investigations, rodents, guinea pigs and rabbits are preferred in early-stage efficacy studies. Testing for ocular hypotensive activity is usually performed in normal eyes with normal physiological IOPs. However, in order to mimic the human OHT/glaucoma condition, animals with experimentally or genetically induced (Harada et al., 2019) OHT are utilized. Thus, mice, rats, rabbits, monkeys, dogs, cats, pigs, guinea pigs, sheep, and cattle have been used to determine the relative IOP-lowering efficacy of topical ocularly administered test agents (Johnson and Tomarev, 2010; Stewart et al., 2011; Bouhenni et al., 2012; Iglesias et al., 2015; Ishikawa et al., 2015; Agarwal and Agarwal, 2017; Biswas and Wan, 2019; Shah et al., 2019). Ideally, naturally occurring animal models of human OHT/glaucoma should be used but unfortunately only limited and costly versions of such animals exist which include, for example DBA/2J mice (pigmentary glaucoma; Williams et al., 2013), beagle dog (congenital glaucoma) (Palko et al., 2016) and wild monkeys (Widdig et al., 2016). However, Dutch-belt rabbits naturally have high IOPs and are good responders to most drugs, especially when intravitreally injected (e.g., Sharif et al., 2015). Thus, initial screening of test compounds for IOP-lowering efficacy can be conducted in rodents or rabbits using a vehicle control and if possible a positive control on a frequent basis to verify the fidelity of the primary screening model(s). Compounds of high interest can then progress to the secondary (e.g., normotensive monkey eyes) and tertiary (OHT monkey eyes) animal models and ocular hypotensive activity determined.

**FIGURE 17:**
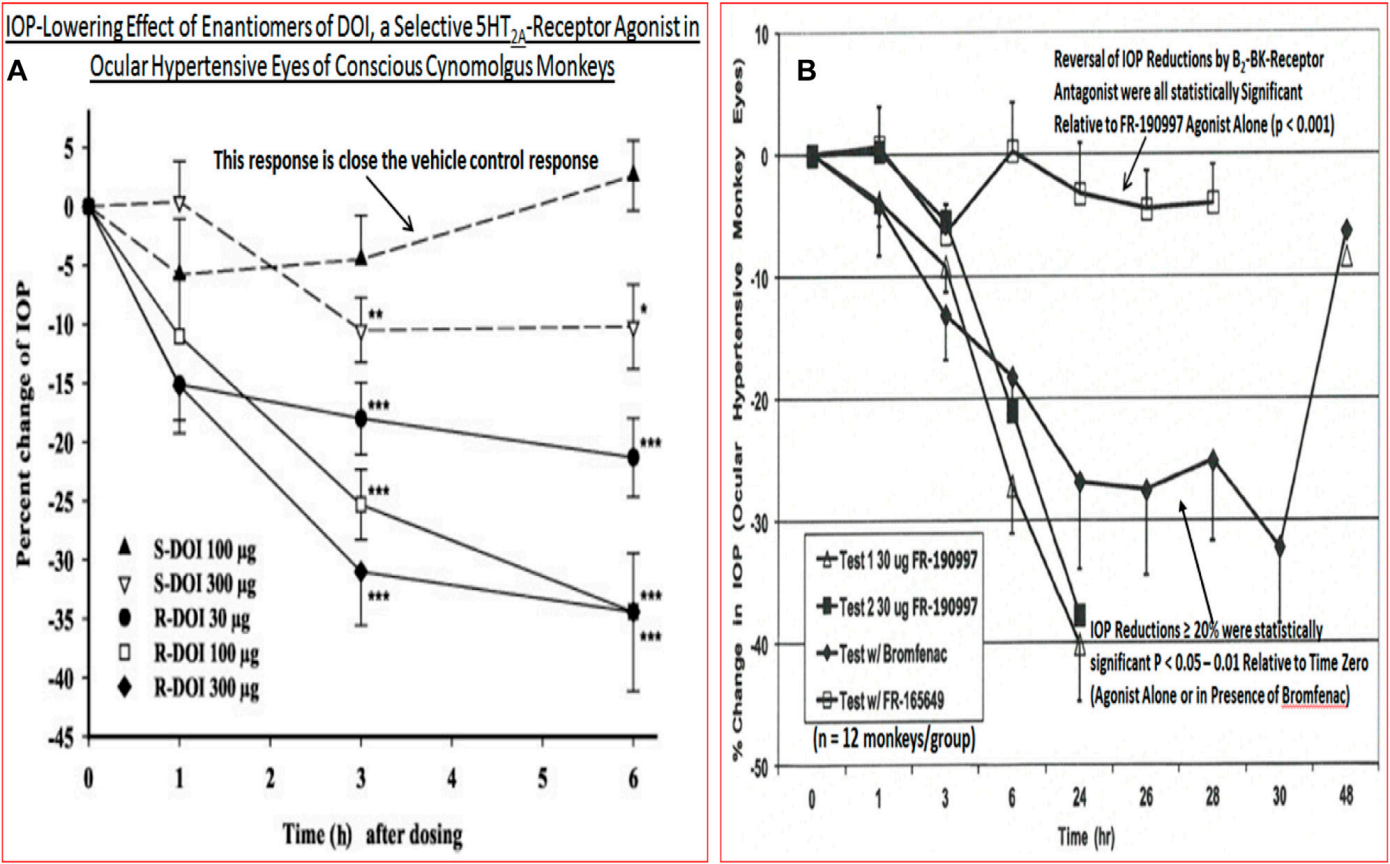
The ability of two different classes of compounds (S- and R-DOI, a 5-HT2 receptor agonist **(A)** and a non-peptidic bradykinin B2-receptor agonist [FR-190997] **(B)**) to lower IOP in conscious ocular hypertensive Cynomolgus monkey eyes is shown.

OHT eyes of rodents and rabbits can be created using a variety of techniques using hypertonic saline injection into the episcleral veins (Morrison et al.), photocoagulation of these veins (Shareef et al., 1995), injection of microbeads (latex (Sappington et al., 2010; Yang et al., 2011) or magnetic (Samsel et al., 2011; Ito et al., 2016)), or by other techniques including intermittent IOP elevation (Gramlich et al., 2016), or by injecting a viscoelastic material (Cone et al., 2010) into the ANC of the eye. Monkey eyes can be rendered OHT by lasering of a large portion of their TM which leads to a fairly reproducible elevated IOP within a range of 30–40 mmHg. To reduce injury to the eye, a mild anesthetic (proparacaine) is used to partially numb the ocular surface to permit the measurement of the IOPs using various pneumotonometer devices (TonoLab; Tono-Pen, Goldman pneumotonometer; Pease et al., 2000; Millar et al., 2015) to monitor and ensure stable IOP-elevations are achieved (Hellberg et al., 2002; May et al., 2003; May et al., 2006; Sharif et al., 2007; Sharif et al., 2014a; Sharif et al., 2014b). The contralateral eye is not operated on and represents a normotensive control eye. Even though these non-human primate models of OHT/glaucoma are stable over many years and are fairly predictive of efficacy in glaucoma patients, there are always exceptions and many surprises in terms of the magnitude of the response and its time-course. Hence, translatability and extrapolation of ocular hypotensive actions of different classes of drugs from the monkey model to the human glaucomatous situation requires a cautious approach. Similarly, confirmation of the IOP-lowering results in multiple colonies of animals (e.g., Sharif et al., 2014a; Sharif et al., 2014b), and if possible at multiple research facilities (Sharif et al., 2014a; Sharif et al., 2014b), with the compound testing performed in a fully masked/coded manner, is highly recommended. Moreover, after suitable washout periods and rest, the monkey eyes in different colonies should be evaluated with multiple positive control ocular hypotensive agents to ensure continued responsiveness to different classes of compounds. Some examples of ocular hypotensive efficacy of two different compounds (R-DOI (5-HT2 receptor agonist) and FR-190997 (non-peptidic bradykinin B2-receptor agonist) in eyes of fully trained conscious and seated cynomolgus monkeys are displayed in Figures 17A,B.

If a high capacity screening model is desired for testing a large number of potential IOP-lowering agents then rodents are probably the best animal model. They are relatively cheap, easier to handle, cheaper to maintain and breed. The similarity of the mouse eye structure and genomics to humans are additional benefits (Stewart et al., 2011; Bouhenni et al., 2012; Iglesias et al., 2015; Ishikawa et al., 2014; Agarwal and Agarwal, 2017). Furthermore, rodent eyes can be injected with gene-delivery systems to help express (or genetically delete certain genes and gene-products) that have been linked to the development of OHT/POAG (e.g., Shepard et al., 2010). These engineered mice, along with naturally occurring glaucomatous mice of the DBA/2J strain (Libby et al., 2005; Howell et al., 2007; Fernandes et al., 2013; Fernandes et al., 2014) and YBR/EiJ (Nair et al., 2016) strain, or steroid-induced glaucoma mouse model (Maddineni et al., 2020) for example, have been valuable in the study of OHT/GON disorder. Similarly, gene knock-out mice have been vital in elucidating the involvement of certain receptors mediating ocular hypotension. For example, FP-receptor agonists such as travoprost, latanoprost and bimatoprost robustly lowered IOP in wild-type mice but failed to show any efficacy in the FP-receptor-knock-out mice (Ota et al., 2005; Ota et al., 2007). Such studies have confirmed that all these PGs produce their ocular hypotensive effects by stimulating the same common FP-receptor (Maxey et al., 2002; Camras et al., 2008), despite claims of bimatoprost working through an enigmatic “prostamide-receptor” (Burk and Woodward, 2007).

In order to help differentiate ocular hypotensive compounds, mechanistic studies have proven useful. The glaucoma monkey model (e.g., Sharif et al., 2014a) and a mouse model (Millar et al., 2011) have been utilized to study AQH dynamics to ascertain whether compounds lower IOP by stimulating AQH outflow via the TM/SC and/or UVS pathways or by inhibiting AQH production. Studies conducted on anterior segments of bovine, porcine, murine and human eyes (Llobet et al., 1999; Crosson et al., 2005; Webb et al., 2006; Keller et al., 2008; Mao et al., 2011; Boussommier-Calleja et al., 2012; Ren et al., 2016) have also been useful to extend observations made in the intact eyes of living animals (see above). The latter *ex-vivo* models lend themselves to further mechanistic investigations since various enzyme inhibitors, receptor antagonists or other drugs can be perfused and their effects studied on the outflow parameters. Some examples of compounds tested recently in such *ex-vivo* models that stimulated outflow include a non-peptide bradykinin-mimetic (FR-190997), and a mixed dopaminergic and 5HT_2_ receptor agonist (cabergoline) (Figure 16B). One study showed that bradykinin caused outflow enhancement in a bovine anterior segment model by releasing endogenous MMP-9, and that this effect could be blocked by a B_2_-receptor antagonist (Webb et al., 2006).

## Need for Neuroprotection in Glaucoma Treatment

Even though NTG patients have IOPs in the normal range (16–21 mmHg), they continue to experience vision loss. Also, it is estimated that 30–80% of OHT/glaucoma patients’ disease continues to progress despite adequate IOP reduction. Conversely, many humans have abnormally elevated IOPs but they do not progress to GON and their vision is unaffected by the raised IOP. These observations strongly suggest that either IOP-independent events and pathologies underlie the development of GON in the NTG patients or that their visual system components are much more sensitive to the IOP levels which are considered “normal”. The same may apply to other patients with other forms of glaucoma. Indeed, accumulating evidence indicates that OHT alone is not the sole contributor to glaucomatous damage and the resultant visual impairment as discussed earlier. OHT and other damaging events ongoing simultaneously probably conspire to cause visual impairment in NTG/POAG/PACG patients. Thus, IOP-independent mechanisms involving retinal vascular dysfunctions (Flammer and Orgul, 1998; Broadway and Drance, 1998; Cull et al., 2015; Pasquale, 2016) and the accompanying oxidative stress (Tezel, 2006; Nickells et al., 2012; Peters et al., 2015) and RGC/axonal energy depletion due to mitochondrial defects (Osborne, 2010; Osborne et al., 2016; Li et al., 2015; Chaphalkar et al., 2020) appear to be culprits. As discussed in the earlier section above, local inflammation at the ONH/LC due to release of injurous cytokines/chemokines/proteases (Albalawi et al., 2017) and at the RGC dendritic/soma sites are responsible for destroying the components of the optic nerve and the RGCs and other retinal interneurons. Excessive release of cellular ATP from dying cells leads to further damage to neighboring cells through activation of the purinergic receptors on RGCs and other retinal neurons through the generation of inflammasomes (Romano et al., 2020). Furthermore, recent research has shown that retrograde axonal flow of mitochondria and neurotrophins (Iwabe et al., 2007) from the brain to the RGC somas becomes defective with OHT (Gaasterland et al., 1978; Radius and Anderson, 1981; Pease et al., 2000; Band et al., 2009; Crish et al., 2013; Ito and Di Polo, 2017) and these changes negatively impact the overall RGC and optic nerve health. Additionally, defects in the generation and maintenance of sufficient levels of intracellular ATP by mitochondria and/or deficits in nicotinamide adenine dinucleotide (NAD^+^) or its congeners, and/or abnormal mitochondrial metabolism/catabolism within retinal neurons of patients with NTG/POAG may heavily contribute to their GON and visual deficits (Thomas et al., 2000; Barron et al., 2004; Bessero and Clarke, 2010). Hence, direct protection of RGCs, their axons and the ONH architecture from such IOP-dependent and IOP-independent insults is necessary in addition to further reducing the patients’ IOPs.

Significant progress has been made in the discovery and characterization of preclinically effective neuroprotectants/cytoprotectants at least from *in vitro* assay systems using a variety of animal and human isolated retinal, LC and ONH cells subjected to various chemical and mechanical stressors (see He et al., 2018; Sharif, 2018a; Sharif, 2018b; Sharif, 2020a; Sharif, 2020b for reviews). Additional supportive data for the neuroprotective activity of numerous classess of drugs and treatment modalities have been gathered using various animal models of GON (Sharif, 2018a; Sharif, 2018b). The burgeoning list of cyto-/neuro-protective agents include anti-oxidants (Aksar et al., 2015), B-vitamin supplements and other nutriceuticals (Luna et al., 2012; Davis et al., 2017; Williams et al., 2017a; Williams et al., 2017b; Saccà et al., 2019; Cammalleri et al., 2020; Chou et al., 2020), antagonists of excitotoxic glutamate receptors (Sharif et al., 2001; Ju et al., 2015; Opere et al., 2018), valproic acid (Kimura et al., 2015a; Kimura et al., 2015b), Ca^2+^-channel blockers, anti-inflammatory agents/glial cell modulators (Cueva Vargas et al., 2016), ATP-sensitive K^+^-channel openers, neurotrophic compounds (Hu et al., 2005; Khatib and Martin, 2017), neurosteroids (Ishikawa et al., 2014), α2-adrenoceptor agonists, anti-epileptics, nicotininc receptor agonist (Iwamoto et al., 2014), mTOR inhibitors, Janus kinase inhibitors, MAP kinase inhibitors, delta opioid receptor agonists, endothelin antagonists, sigma-1 receptor agonists (Ellis DZ. et al., 2017), etc., etc (recently reviewed by He et al., 2018; Sharif, 2018) which are effective ameliorators of cell death of RGCs/LC cells/TM cells, and which also possess *in vivo* efficacy in various animal models of ischemia/hypoxia/oxidative stress/neurotrophin- and glucose-deprivation/direct excitotoxicity/optic nerve crush or transection emulating what may be happening in the human GON condition in patients with NTG/POAG (see He et al., 2018; Sharif, 2018 for reviews). Delivery of mitochondrial-targeted anti-oxidants (Pang et al., 2015), inactivation of the NFkB system in astroglia (Dvoriantchikova et al., 2009; Dvoriantchikova and Ivanov, 2014), use of inducible NO synthase inhibitors (Schnichels and Joachim, 2021), RGC and/or stem cell transplatation in the retina (Johnson and Martin, 2013; Behtaj et al., 2020; Liu and Lee, 2021), migroglial removal and repopulation (Barnett et al., 2021), delivery of a gene-therapy-based neurotrophin receptor-ligand complex (Khatib et al., 2021), and direct delivery of Schwann cells to the optic nerve also represent promising approaches to treat GON (Guo et al., 2014; Smedowski et al., 2016). Directly addressing mitochondrial defects/deficiencies using mitofusin activators (Rocha et al., 2018; Dang et al., 2020) may support mitochondrail rescue and help in axonal regeneration (Zhou et al., 2016).

Of the drugs that have been clinically evaluated for neuroprotective activity and to retard vision loss or impairment in POAG patients, for example brimonidine and memantine, none have conclusively proven effective thus far (Almasieh and Levin, 2017; Levin, 2017). It is worth noting that a very recent study demonstrated the neuroprotective activity of brimonidine after retinal ischemia (Conti et al., 2021). There is some hope and promise that oral vitamin-B3/nicotinamide may offord some degree of functional vision preservation in POAG patients (Williams et al., 2017c; Hui et al., 2020). Likewise, the potential utility of senolytic drugs such as quercetin, nicotinib, imatinib, toclizumab and dasatinib (El-Nimri et al., 2020) that help remove dead RGCs and neuroprotect may become commonly used therapeutics to treat POAG/NTG in addition to using IOP-lowering drugs. As with many reports of this kind, these results need to tbe confirmed by other researchers in different parts of the world in multiple additional clinical trials. Moreover, given the multiplicity of deleterious factors and conditions that trigger and sustain neuronal/axonal injury and demise (see above), and the gross heterogeniety of RGC-types (Sanes and Masland, 2015; Ou et al., 2016; Della Santina and Ou, 2017; Tran et al., 2019) with varying degrees of susceptibility to or resiliency to damage, it is likely that a combinatorial approach to mitigate and stem the vision damage/loss from GON caused by different forms of glaucoma would be necessary. This poses significant hurdles from a regulatory perspective to conduct suitable clinical trials and to gain approval from health agencies around the world. Nevertheless, a concerted collaborative effort by researchers and health autority personnel could prove productive in order to help save the eyesight of millions of patients afflicted with GON due to POAG/PACG/NTG. Hence, successful clinical translation of the neuroprotection paradigm in the glaucoma patients is eagerly awaited (Boia et al., 2020), which may also involve the use of suitable stem cells (Chamling et al., 2016) and/or their secretome (Harrell et al., 2019), intravitreally injected Schwann cells and potential optic nerve regeneration (Li et al., 2004; Li et al., 2017), chimeric perpheral nerve grafts (Cui et al., 2003).

## Conclusion

**FIGURE 18:**
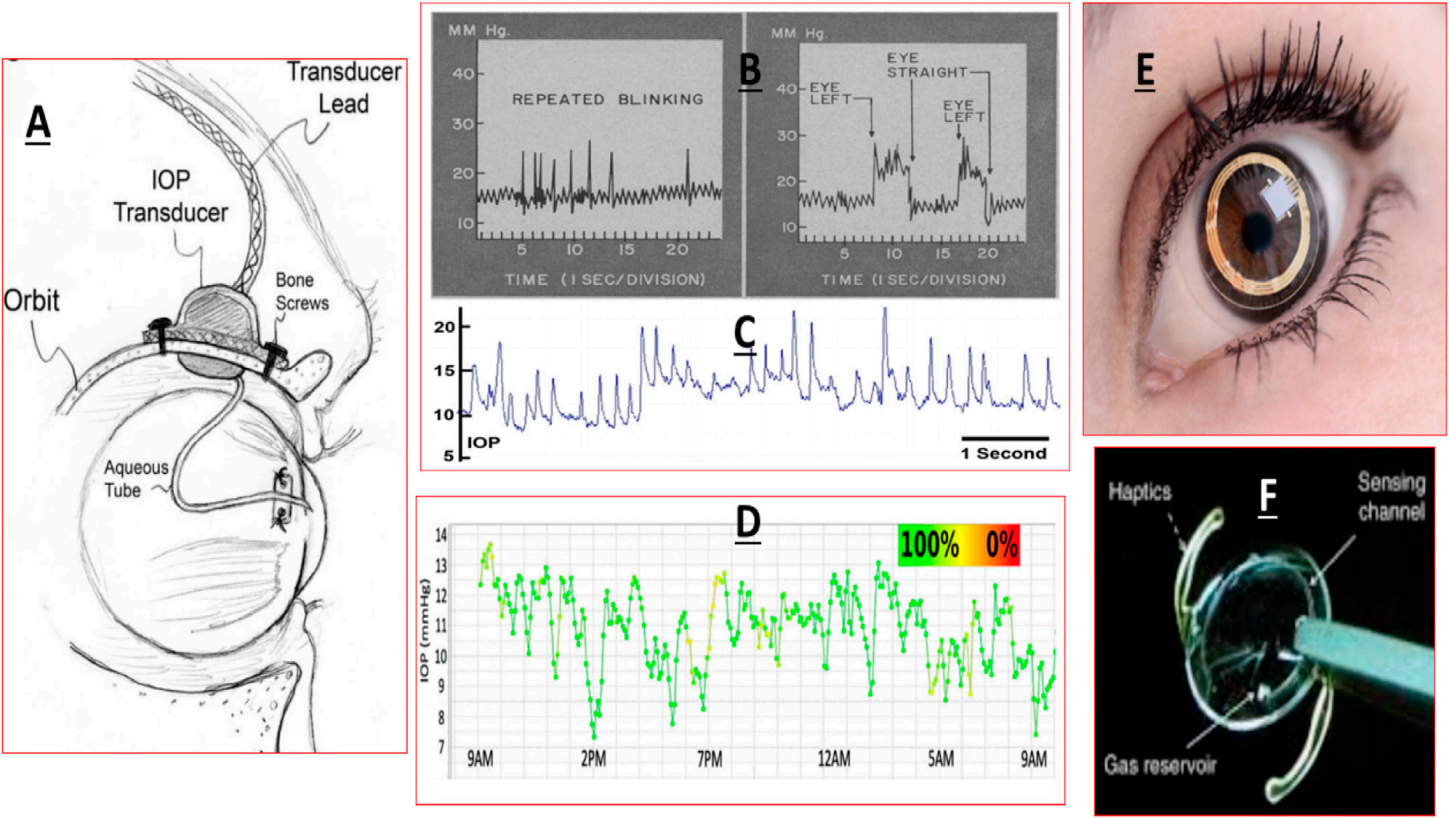
Examples of use of remote telemetric monitoring of IOP in conscious monkey eyes **(A–C)**, in human eyes with a contact lens-based device **(D,E)** and an intraocular lens-bearing device **(F)** are shown.

The above discourse provides strong evidence for involvement of defective anterior chamber physiology in the onset of POAG, PACG and other forms of glaucoma. Specifically, the aging process leads to energy depletion in mitochondria (Kleesattel et al., 2015; Eells, 2019) and cellular debris accumulation in the corneoiridial angle of the ANC. Such TM occlusion coupled with reduced TM cellularity, phagocytotic activity and flexibility in the conventional outflow pathway directly contribute to the increased resistance to AQH outflow causing elevation of IOP. This OHT-induced mechanical pressure leads to a cascade of detrimental events, including inflammation (Slater et al., 2013), at the retinal ONH/LC and optic nerve regions which ultimately causes RGC axonal damage and RGC death resulting in vision loss, especially resulting in peripheral vision defects (Tribble et al., 2020). Several strategies to pharmaceutically and surgically (including use of implanted microshunts) reduce IOP have been developed to treat OHT. However, since effective IOP control does not prevent visual impairment in many patients suffering from glaucoma, especially those with NTG who have normal IOPs (Jampel, 2007), the notion that IOP-independent mechanisms cause GON is now well accepted. Therefore, future treatment modalities for glaucoma will necessitate the use of neuroprotectants and neuroregenerative paradigms whilst still controlling the IOP (Goldberg, 2012; Guymer et al., 2019; Tsai, 2020). Several classes of pharmaceutical agents/nutriceuticals and regenerative compounds have demonstrated efficacy in cell-based assays and in a number of *in vivo* models of GON as described above (Goldberg, 2012; Guymer et al., 2019; El-Nimiri et al., 2020). However, translation of such efficacy in glaucoma patients needs to be demonstrated in multiple clinical trials on a worldwide basis to help save eyesight for millions of people around our planet. It is also imperative that high priority be assigned to early diagnosis of the glaucomatous conditions so that patients begin their treatments to slwo down their visual impairment. The development of innovative imaging techniques such as those using nucleic acid dyes (Tsuda et al., 2016) and DARC (detecting apoptosizing retinal cells; Cordeiro et al., 2017), adaptive optics (Bower et al., 2021), angiography-coupled optical coherance tomography (Kaizu et al., 2017), and of course magnetic resonance imaging (MRI; Chow and Paley, 2021) should aid in early diagnosis and quantification of the damage to retinal neurons/optic nerve with and without neuroprotective treatment modalities. Likewise, the future appears bright for use of gene therapy and cell-therapy to potentially correct genetic or disease-induced abnormalities in TM/SC and retinal cells (Khatib and Martin, 2017; Ratican et al., 2018). Perhaps replacing or adding new healthy cells to the ANC and retina, and/or operationalizing gain of function of the latter by normalizing metabolic defects at the mitochondrial level of these cells with appropriate food supplementation or direct injection to the vitreous in sustained delivery vehicles may be possible. Direct repair and/or protection of RGC bodies and dendrites, and the myelin sheath and other components of the RGC axons (Fang et al., 2010), regenration of the optic nerve projecting to the brain (Van de Velde et al., 2015), also hold great promise. Normalization of intracranial fluid pressure to help physically support the optic nerve is potentially another treatment option in the future for patients whose fluid dynamics at the ANC and intracranially become defective due to the aging process or induced by disease mechanisms as discussed above. Ability to monitor IOP round the clock using appropriate sensors (De Moraes et al., 2018; e.g., Figure 18) will offer clinicians tools to diagnose POAG earlier, and perhaps patients to self-evaluate their eye health and seek treatment early as well. Similarly, the role of artificial intelligence in drug discovery, dignosis of POAG, and other aspects of ocular disease treatment represents a novel platform to increase awareness/diagnosis of glaucoma (Zheng et al., 2019).

## Author Disclaimer

The author is an employee of Santen Inc. (United States) and an Adjunct Professor at multiple global Universities, and declares no conflict of interest. The author’s intentions and goal are simply to advance knowledge and help disseminate information that will help further the research efforts in ocular pharmacology and ophthalmic therapeutics development to treat eye diseases, especially glaucoma and GON. As an Adjunct Professor at multiple global universities and learned institutions, it has been a pleasure to collaborate with many professors and their team members over the years, and I have thoroughly enjoyed working with, teaching and mentoring many undergraduate and graduate students over the course of my career. It is hoped that the author’s contributions have had a beneficial effect on the students’ learning, their self-esteem and their future career prospects, and that my efforts are meaningful and helpful to the ocular research community and the scientific community at large. It is regretable that time and space constraints do not permit citation of so many other researchers’ publications and it is hoped that the reader will follow-up with further literature searches as deemed fit.

## Abbreviations

AMD, age-related macular degeneration; ANC, anterior chamber; AQH, aqueous humor; IOP, intraocular pressure; PG, prostaglandin; RGC, retinal ganglion cell; TM, trabecular meshwork; SC, Schlemm’s canal; OHT, ocular hypertension; LC, lamina cribosa; ONH, optic nerve head; CRA, central retinal artery; SPCA, short posterior ciliary artery; CRV, central retinal vein; CB, ciliary body; CP, ciliary processes; CE, ciliary epithelium; CM, ciliary muscle; JXTC, juxtacanalicular; RPE, retinal pigmented epithelium; MGC, Muller glial cell; LGN, lateral geniculate nucleus; SC, superior colliculus; VC, visual cortex; VH, vitreous humor; NPCEC, non-pigmented ciliary epithelial cell; UVS, uveoscleral; PACG, primary angle-closure glaucoma; POAG, primary open angle glaucoma; GFAP, glial fibrillar acid protein.
